# Genomic analysis of methanogenic archaea reveals a shift towards energy conservation

**DOI:** 10.1186/s12864-017-4036-4

**Published:** 2017-08-21

**Authors:** Sean P. Gilmore, John K. Henske, Jessica A. Sexton, Kevin V. Solomon, Susanna Seppälä, Justin I Yoo, Lauren M. Huyett, Abe Pressman, James Z. Cogan, Veronika Kivenson, Xuefeng Peng, YerPeng Tan, David L. Valentine, Michelle A. O’Malley

**Affiliations:** 1 Department of Chemical Engineering, University of California, Santa Barbara, California, USA; 2 Novo Nordisk Foundation Center for Biosustainability, Technical University of Denmark, Hørsholm, Denmark; 3 Biology Program, College of Creative Studies, University of California, Santa Barbara, California, USA; 4 Department of Earth Science and Marine Science Institute, University of California, Santa Barbara, California, USA; 5 California NanoScience Institute, University of California, Santa Barbara, California, USA; 6 Present Address: Agricultural & Biological Engineering, Purdue University, West Lafayette, Indiana, USA

**Keywords:** Methanogenesis, Archaea, Metabolism, Anaerobes, Energy

## Abstract

**Background:**

The metabolism of archaeal methanogens drives methane release into the environment and is critical to understanding global carbon cycling. Methanogenesis operates at a very low reducing potential compared to other forms of respiration and is therefore critical to many anaerobic environments. Harnessing or altering methanogen metabolism has the potential to mitigate global warming and even be utilized for energy applications.

**Results:**

Here, we report draft genome sequences for the isolated methanogens *Methanobacterium bryantii*, *Methanosarcina spelaei*, *Methanosphaera cuniculi*, and *Methanocorpusculum parvum*. These anaerobic, methane-producing archaea represent a diverse set of isolates, capable of methylotrophic, acetoclastic, and hydrogenotrophic methanogenesis. Assembly and analysis of the genomes allowed for simple and rapid reconstruction of metabolism in the four methanogens. Comparison of the distribution of Clusters of Orthologous Groups (COG) proteins to a sample of genomes from the RefSeq database revealed a trend towards energy conservation in genome composition of all methanogens sequenced. Further analysis of the predicted membrane proteins and transporters distinguished differing energy conservation methods utilized during methanogenesis, such as chemiosmotic coupling in *Msar. spelaei* and electron bifurcation linked to chemiosmotic coupling in *Mbac. bryantii* and *Msph. cuniculi*.

**Conclusions:**

Methanogens occupy a unique ecological niche, acting as the terminal electron acceptors in anaerobic environments, and their genomes display a significant shift towards energy conservation. The genome-enabled reconstructed metabolisms reported here have significance to diverse anaerobic communities and have led to proposed substrate utilization not previously reported in isolation, such as formate and methanol metabolism in *Mbac. bryantii* and CO_2_ metabolism in *Msph. cuniculi*. The newly proposed substrates establish an important foundation with which to decipher how methanogens behave in native communities, as CO_2_ and formate are common electron carriers in microbial communities.

**Electronic supplementary material:**

The online version of this article (doi:10.1186/s12864-017-4036-4) contains supplementary material, which is available to authorized users.

## Background

Methanogenic archaea are key players in anaerobic communities and as such contribute largely to global warming and energy production, with interesting potential roles in human and animal health [1, 2]. These extraordinary microbes perform a type of anaerobic respiration known as methanogenesis by reduction or dismutation of carbon dioxide, methyl compounds, or acetate to methane or methane and carbon dioxide in several ecosystems and consortia [3]. Methanogenesis operates at a very low reducing potential compared to other forms of aerobic and anaerobic respiration [4]. As such, methanogens can be found in such diverse environments as the deep ocean, rice paddies, wetlands, landfills, and the gastrointestinal tracts of termites, ruminants, and humans, where more favorable electron acceptors like oxygen or inorganic ions are unavailable [1]. Because they constitute a major source of atmospheric methane, there has been a drive to increase understanding of methanogen metabolism and their interactions with other organisms [5]. This understanding of methanogen metabolism is necessary for optimal design of biochemical processes aimed at generating or removing methane [6, 7].

Many studies seek to mitigate negative effects contributed by methanogens. Livestock, as a component of agriculture, are the largest anthropogenic source of atmospheric methane [8]. Certain methanogens thrive in the rumen compartment of cattle, where they partner with cellulose-degrading gut microbes, consuming the hydrogen and carbon dioxide these microbes produce as waste [9]. This phenomenon, known as enteric emission, produces approximately 30% of global methane emissions, a greenhouse gas 25 times more potent than carbon dioxide [5, 8]. The effect of methanogens on human and animal health is less clear. Although there are conflicting reports, many studies describe a direct correlation between the presence of methanogens in human guts and the occurrence of diseases such as periodontitis, obesity, and inflammatory bowel disease [2].

The high energy content of methane has recently called attention to methanogens as a tool in waste-to-energy technologies [7, 10]. As in the rumen and other natural anaerobic environments, methanogens can partner with other microbes in specialized anaerobic reactors to convert organic biomass into methane [11]. This process, termed anaerobic digestion, results in generation of potentially-useful biogas [11]. Recently, anaerobic microbes have received attention for their ability to thrive on crude biomass [12–14]. Methanogens enable the growth of other microbes by removing self-limiting waste products, which methanogens assimilate or use as electron donors/acceptors in methanogenesis [3, 15]. For this reason, the substrate utilization capabilities of methanogenic species are of great interest, as is elucidating the details of ATP generation via carbon dioxide reduction. Comparing and contrasting the metabolic pathways in methanogenic species of diverse evolutionary origins will be vital to progress in this area.

Currently, methanogens are grouped in three ways: by energy requirements, based on phylogeny, or by catalytic abilities. Metabolic grouping depends on energy source, which can be i) hydrogen/formate and carbon dioxide, ii) methyl compounds, or iii) acetate [1]. Phylogenetic grouping based on 16S rRNA sequencing comprises only two classes: Class I and Class II [16]. This classification also reflects metabolic differences, with basal Class I lineages requiring hydrogen and carbon dioxide, and sometimes also formate, and the more recently evolved Class II lineages requiring either methyl compounds or acetate [17, 18]. Recently, Anderson et al. conducted a new type of phylogenetic analysis and proposed that Class II be defined to include only species of the Methanomicrobiales order, while the Methanosarcinales would form a new class: Class III [18]. Recently, new genomes and metagenomes sequenced have identified new clades of methanogens outside of the early-established class systems, such as the *Methanomassiliicoccales* [19] or the newly discovered *Methanonatronarchaeia* [20].

We sought to better understand metabolic capabilities of methanogens and the biochemical pathways behind them. To this end, we obtained four methanogenic species from diverse environments and sequenced their genomes. Under the three class system [18], *Methanobacterium bryantii* and *Methanosphaera cuniculi* are Class I methanogens requiring H_2_/CO_2_ and H_2_/methanol, respectively. *Methanocorpusculum parvum* is a member of Class II and requires H_2_/CO_2_, formate, or 2-propanol/CO_2_. Finally, *Methanosarcina spelaei* is a member of Class III and utilizes H_2_/CO_2_, acetate, or methyl compounds for growth and methanogenesis. By sequencing the genomes of each member and reconstructing their metabolisms, we seek to elucidate the metabolic pathways and mechanisms implemented by each of the three classes and their contributions to energy, health, and agriculture.

## Methods

### Methanogen culture and DNA isolation

*Methanobacterium bryantii* strain M.o.H. (DSM 863), *Methanosarcina spelaei* strain MC-15 (DSM 26047), *Methanosphaera cuniculi* strain 1R-7 (DSM 4103), and *Methanocorpusculum parvum* strain XII (DSM 3823) were ordered from the Leibniz Institute DSMZ – German Collection of Microorganisms and Cell Culture (https://www.dsmz.de/). Cultures were grown in a modified version of M2 medium adapted from Teunissen et al. [21]. The base medium contained 150 mL of Solution A (0.45 g/L KH_2_PO_4_, 0.45 g/L (NH_4_)_2_SO_4_, 0.9 g/L NaCl, 0.09 g/L MgSO_4_·7 H_2_O, 0.09 g/L CaCl_2_·2 H_2_O), 150 mL of Solution B (0.45 g/L K_2_HP0_4_), 12 g/L NaHCO_3_, 1 mL of Vitamin Supplement (ATCC MD-VS) supplemented with 0.9 μg cyanocobalamin and 40 mg CoM, 10 mL trace element solution (2.5 mg/mL MnCl_2_·4H_2_O, 2.5 mg/mL NiCl_2_·6H_2_O, 2.5 mg/mL NaMoO_4_·2H_2_O, 2.5 mg/mL H_3_BO_3_, 2.0 mg/mL FeSO_4_·7H_2_O, 0.5 mg/mL CoCl_2_·6H_2_O, 0.5 mg/mL SeO_2_, 0.5 mg/mL NaVO_3_·4H_2_O, 0.25 mg/mL ZnCl_2_, 0.25 mg/mL CuCl_2_·2H_2_O, all dissolved in 0.2 M-HCl), 10 mL hemin solution (1 mg/L hemin, 0.01% (*v*/v) ethanol, dissolved in 0.05 M NaOH), 1 mL of resazurin solution (0.1% (*w*/*v*) resazurin), 1 g L-cysteine-HCl [22]. The total volume of the medium was adjusted to 1 L with MilliQ H_2_O, boiled vigorously to drive out the oxygen, cooled under CO_2_, and aliquoted under an 80/20 mixture of H_2_/CO_2_ before autoclaving. Vitamin solution and methanol were filter sterilized and added after autoclaving. *Mbac. bryantii* medium was supplemented with 2 g/L sodium acetate, 4 g/L sodium formate, 2 g/L yeast extract, and 4 g/L bacto casitone. *Mcor. parvum* medium was supplemented with 2 g/L sodium acetate, 4 g/L sodium formate, 2 g/L yeast extract, 4 g/L bacto casitone, and 10 μM sodium tungstate. *Msar. spelaei* medium was supplemented with 4.5 g/L sodium chloride, 2 g/L sodium acetate, 2 g/L yeast extract, 2 g/L bacto casitone, and 0.5% (*v*/v) methanol. *Msph. cuniculi* medium was supplemented with 3.6 g/L sodium acetate, 2 g/L yeast extract, 2 g/L bacto casitone, and 1% (*v*/v) methanol. All cultures were grown at 39 °C without shaking. Experiments testing growth on substrates alternative to H_2_ were conducted in 100% CO_2_ headspace.

To isolate genomic DNA, 1 mL of methanogens was inoculated into 40 mL of media in 60 mL Wheaton serum bottles until stationary phase (OD_600_ ~ 0.2–0.5) and then harvested by centrifugation for 30 min at 10,000×g at 4 °C. Cell pellets were resuspended in 0.5 mL TE Buffer (10 mM Tris, 1 mM EDTA, pH 8.0). Sodium dodecyl sulfate was added to a final concentration of 0.5%, proteinase K (New England BioLabs, Ipswitch, MA) was added to 100 μg/mL, and RNaseA (MoBio Laboratories, Carlsbad, CA) was added to 100 μg/mL. The mixture was incubated at 37 °C for 1 h. NaCl was added to 0.5 M, and 0.5 mL of phenol:chloroform:isoamyl alcohol (25:24:1) was added. The solution was mixed and then centrifuged at 13,000×g for 10 min at 4 °C. The aqueous phase was transferred to a new tube and 0.6 mL of isopropyl alcohol was added. The mixture was incubated at −20 °C for ~16 h and then centrifuged at 13,000×g for 5 min at 4 °C. The pellet was washed with 70% ethanol, centrifuged at 13,000×g for 5 min at 4 °C, and finally resuspended in 10 mM Tris buffer pH 8.0 and stored at −20 °C [17].

### Library preparation and sequencing

Genomic DNA (gDNA) was prepared for high throughput sequencing (HTS) using the TruSeq DNA PCR-Free library prep kit supplied by Illumina, Inc. (San Diego, CA). Briefly, purified gDNA was first fragmented using a Covaris (Woburn, Massachusetts) M220 Focused Ultrasonicator, followed by end repairs, size selection (~330 bp), end adenylation and paired-end adapters ligation using the kit. Prepped libraries were then quantified using Qubit (Life Technologies, Carlsbad, CA) and TapeStation (Agilent, Santa Clara, CA), before pooling. HTS was performed with an Illumina NextSeq500 sequencer using a 150 cycle, mid output kit (2 × 75 paired-end). Resequencing of *Methanosarcina spelaei* was completed using a 75 cycle mid output kit (75 bp single-end).

### Genome assembly and annotation

Genomes were assembled from 75 bp paired end Illumina NextSeq reads (Illumina, San Diego, CA) at a minimum of 49× coverage and annotated with the Department of Energy Systems Biology Knowledgebase (KBase, http://kbase.us) automated pipeline. Briefly, reads were preprocessed with BayesHammer [23] before being assembled de novo into contigs with the SPAdes and Velvet genome assembly algorithms [24, 25]. Genomic features including ORFs, large repeat regions, rRNAs, CRISPRs, and tRNAs were then identified and annotated with the Rapid Annotations using Subsystems Technology toolkit (RASTtk) [26]. These gene annotations were combined with biochemical information from the Kyoto Encyclopedia of Genes and Genomes (KEGG) [27] to reconstruct the metabolism of each methanogen. The assembly for *Methanosphaera cuniculi* contained a number of low coverage (~1X) contigs, which were removed from the draft assembly and analysis. Scaffolding of assembled contigs was performed using SSPACE [28].

### COG analysis

Coding domains were assigned to Clusters of Orthologous Groups (COG) classes by using the RPSBLAST program, version 2.2.26 against the CDD database [29], which was downloaded from the NCBI website on April 24th, 2015. The COG annotations were used to generate genome maps using a combination of BLAST Ring Image Generator (BRIG) [30] and CGview [31]. A random sample of 100 genomes from the RefSeq database [32] was downloaded on July 7th, 2016. The random genomes were assigned to COG classes using the previously described method. To estimate error in the sampling, the 100 genomes were sampled with replacement 1000 times. Significance was determined through Fisher’s exact test, and verified through difference of medians analysis [33]. For comparative purposes, the fraction of genes assigned to a given COG class was defined as the number of genes assigned to the class divided by the total number of genes assigned to any COG class, which accounted for differing genome sizes and unknown gene content.

The evolutionary history of the methanogen isolates was inferred using the Neighbor-Joining method [34]. A sum of branch length of 1.91983843 was used. A bootstrap test with 1000 replicates was used to compute the final tree [35]. The evolutionary distances were computed using the Maximum Composite Likelihood method [36] and are in the units of the number of base substitutions per site. The analysis involved 91 nucleotide sequences. All positions with less than 95% site coverage were eliminated. That is, fewer than 5% alignment gaps, missing data, and ambiguous bases were allowed at any position. There were a total of 1246 positions in the final dataset. Evolutionary analyses were conducted in MEGA7 [37]. Redundant and unnecessary branches were trimmed for a more easily viewed tree and formatted using the Interactive Tree of Life [38].

### Additional annotation and analyses

Possible modes of methanogenesis as described on metacyc.com [39] and in Blaut [40] were investigated for each genome. Using the R programming language, an algorithm was developed to search the functional annotations provided by the KBase pipeline for genes that encode enzymes involved in methanogenesis. Methanogenic genes were identified by searching for either Enzyme Commission (EC) numbers [41] or specific enzyme names and functional descriptions. Hydrogenases were identified by searching the KBase annotations specifically for Eha, Ehb, Mvh, Frh, Ech, Vho, and cytochrome b enzymes. Since these enzymes are multisubunit complexes, the components of each hydrogenase were identified. BLAST-based comparisons were made using BLASTP version 2.2.30+ against the genome indicated, each downloaded from the RefSeq database [32] on August 1st, 2016.

Transmembrane domains were predicted using a Transmembrane Hidden Markov Model (TMHMM) [42]. Transporter analysis was conducted by running BLASTP version 2.2.30+ against the transporter classification database (TCDB) [43], downloaded on January 13th, 2015. A custom python script was used to select the blast hit with the lowest e-value, with the strict requirement that the blast hit matched at least 70% of the length of the subject and query. Membrane proteins and transporters were assigned classes based on TCDB number (transporters) or by manual curation of KBase annotations (membrane proteins).

### Nucleotide sequence accession numbers

The final genome assembly and annotation information is available in the GenBank database with accession numbers PRJNA300714, PRJNA300715, PRJNA300716, and PRJNA300717 for *Mbac. bryantii, Msar. spelaei, Msph. cuniculi,* and *Mcor. parvum,* respectively.

## Results and discussion

### Genome sequencing and assembly

In order to compare methanogen metabolism and methanogenesis across archaeal genera, four methanogens (*Mbac. bryantii, Msar. spelaei, Msph. cuniculi,* and *Mcor. parvum)* were selected for genomic sequencing and analysis. Collectively, the methanogens chosen represent a diverse set of isolates from four different genera and all three classes [18], capable of methylotrophic, acetoclastic, and hydrogenotrophic methanogenesis, as shown in Table 1 [44–48]. Draft genomes for *Mbac. bryantii, Msar. spelaei, Msph. cuniculi,* and *Mcor. parvum* were assembled and annotated through the Department of Energy Systems Biology Knowledgebase platform (KBase), a summary of which can be seen in Table 2. The size of each genome was as expected for methanogens of their respective genera, with *Msar. spelaei* the largest (5.1 Mb), *Mcor. parvum* and *Msph. cuniculi* the smallest (1.7 and 1.9 Mb, respectively), and *Mbac. bryantii* in between (3.5 Mb). *Mcor. parvum* had the highest GC content (51%), while the other three were more AT-rich (~30% GC content). The GC bias is genome-wide, extending to both coding and intergenic regions. An analysis of codon frequency (Additional file 1: Table S1) reflected the GC bias, where *Mcor. parvum* had the lowest stop codon frequency of TAA (42%), compared to 67%, 73%, and 54% for *Mbac. bryantii, Msph. cuniculi,* and *Msar. spelaei,* respectively [49].

**Table 1:** Methanogens Characterized in this Study. Displays a summary of the four methanogens sequenced: *Mbac. bryantii* isolation data from [46, 47], *Msar. spelaei* isolation data from [48], *Msph. cuniculi* isolation data from [45], *Mcor. parvum* isolation data from [44]

	*Methanobacterium bryantii*	*Methanosphaera cuniculi*	*Methanosarcina spelaei*	*Methanocorpusculum parvum*
Current Classification	*Archaea, Euryarchaeota, Methanobacteria, Methanobacteriales, Methanobacteriaceae, Methanobacterium, bryantii*	*Archaea, Euryarchaeota, Methanobacteria, Methanobacteriales, Methanobacteriaceae, Methanosphaera, cuniculi*	*Archaea,* *Euryarchaeota, Methanomicrobia, Methanosarcinales, Methanosarcinaceae, Methanosarcina*, *spelaei*	*Archaea*, *Euryarchaeota*, *Methanomicrobia*, *Methanomicrobiales, Methanocorpusculaceae*, *Methanocorpusculum*, *parvum*
Methanogen Class [18]	Class I	Class I	Class III	Class II
Gram stain	Variable	Gram-positive	Gram-negative	Gram-negative
Cell shape	Rod	Coccus	Sarcina-like coccus	Irregular coccus
Motility	Non-motile	Non-motile	Non-motile	Weakly motile by single flagellum
Sporulation	Nonsporulating	Nonsporulating	Nonsporulating	Nonsporulating
Optimal temperature range	37–45 °C	35–40 °C	33 °C	15–45 °C
Oxygen requirement	Strictly anaerobic	Strictly anaerobic	Strictly anaerobic	Strictly anaerobic
Carbon Assimilation	CO_2_ Autotrophy	Requires Acetate	CO_2_ Autotrophy	Requires Acetate or Yeast Extract
Energy source	H_2_/CO_2_	H_2_/methanol	H_2_/CO_2_, acetate, methanol, monomethylamine, dimethylamine, trimethylamine	H_2_/CO_2_, formate, 2-propanol/CO_2_
Biosafety level	BSL 1	BSL 1	BSL 1	BSL 1
Isolation source	Syntrophic culture isolated from sewage sludge	Intestinal tract of a rabbit	Subsurface sulfurous lake	Anaerobic sour whey digester inoculated with sewage sludge

**Table 2:** Genome Sequencing Statistics for Strains in this Study

Organism	*Mbac. bryantii*	*Msph. cuniculi*	*Msar. spelaei*	*Mcor. parvum*
Number of reads	7,552,398	6,883,020	9,312,063	4,790,894
Read length (bp)	75	75	75	75
Coverage	166	102	137	213
Total length (Mb)	3.5	1.9	5.1	1.7
Largest scaffold (kb)	1030	188	103	255
Number of scaffolds (>1000 bp)	14	29	293	17
GC%	33	28	39	51
N50 (kb)	764	138	37	116
L50	2	6	48	5
Number of unique genes predicted	3526	1658	5913	1844
Genes assigned to COGs	2243	1139	2814	1322
Genes with signal peptides	300	104	596	167
Genes assigned to transporter classification (TCDB)	416	170	556	247
Genes encoding transmembrane helices	934	331	1367	376
# predicted genes (*n* ≥ 0 bp)	3526	1658	5913	1844
# predicted genes (*n* ≥ 300 bp)	2831	1451	3584	1522
# predicted genes (*n* ≥ 1500 bp)	335	228	500	203
# predicted genes (*n* ≥ 3000 bp)	38	41	54	12
Total length (*n* ≥ 1000 bp)	3,463,789	1,930,335	5,029,712	1,709,622

### Analysis of the distribution of COG proteins reveals a shift in genomic composition towards energy conservation

**Fig. 1:**
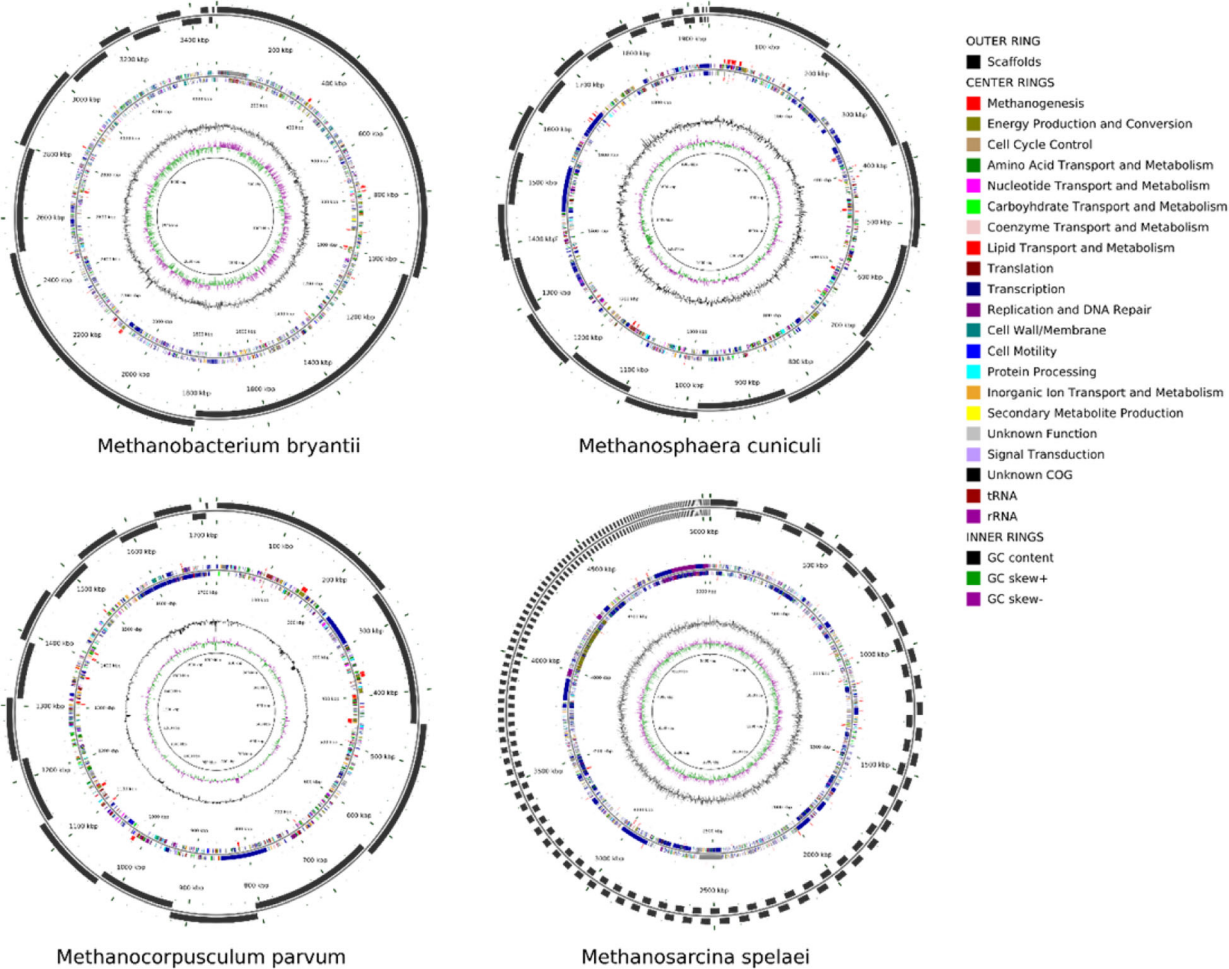
Annotated genome maps highlight key methanogenesis genes. Circular genome maps for *Msar. spelaei, Mbac. bryantii, Mcor. parvum,* and *Msph. cuniculi*. Concentric rings represent the following from outermost to innermost: 1) Assembled Scaffold boundaries. 2) Genes responsible for methanogenesis on the forward strand. 3) Predicted ORFs, colored by COG class, and predicted tRNA and rRNA on the forward strand. 4) Predicted ORFs, colored by COG class, and predicted tRNA and rRNA on the reverse strand. 5) Genes responsible for methanogenesis on the reverse strand. 6) GC Content. 7) GC Skew

The predicted genes were assigned to COG classes using RPSBLAST. A summary of the number of genes assigned to each COG class can be seen in Fig. 1 and Additional file 1: Table S2. The largest classes of COGs represented across the methanogens include Energy Production and Conversion [C], Coenzyme Transport and Metabolism [H], and Inorganic Ion Transport and Metabolism [P]. Analysis of the predicted membrane proteins further demonstrated the trend towards energy conservation (Additional file 1: Figure S1). The three largest classes of membrane proteins and transporters belonged to energy conservation, ion transport, and metal or cofactor acquisition.

**Fig. 2:**
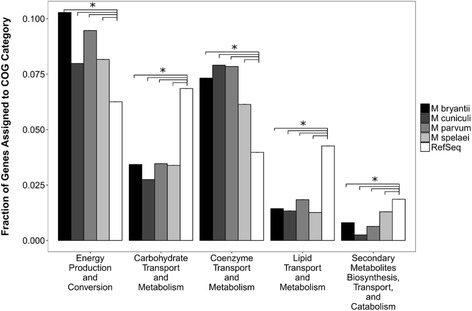
Distribution of COG genes in categories significantly different than the RefSeq Sample. The fraction of proteins in each methanogen belonging to COG categories shown differ significantly compared to the RefSeq sample. All four sequenced methanogens have significantly more genes categorized as energy production and conversion and coenzyme transport and metabolism, which confirms the observation that the methanogens have a confirmed shift towards energy conservation in order to occupy their ecological niche. Significance was determined through Fisher’s exact test, and verified through difference of medians analysis [24], * represents *p* < 0.01

In order to quantitatively compare the COG categories of the sequenced methanogens, a random sample of 100 genomes from the NCBI RefSeq Database was annotated in a similar method. As shown in Additional file 1: Figure S2, the relative distribution of COG proteins from the RefSeq database achieved stability by 100 genomes sampled. Additional file 1: Figure S3 shows a bootstrap analysis of the sampling, which indicates a standard error of less than 0.3% for each COG category distribution. As shown in Fig. 2, all methanogens sequenced were significantly enriched for genes in Energy Production and Conversion (C), Coenzyme Transport and Metabolism (H), and Translation, Ribosomal Structure and Biogenesis (J). Furthermore, they had significantly fewer genes assigned to Carbohydrate Transport and Metabolism (G), Lipid Transport and Metabolism (I), and Secondary Metabolites Biosynthesis, Transport, and Catabolism (Q). The abnormal genome composition, and in particular the enrichment in Energy Production and Conversion genes, has been noted previously [19, 50–52]. As such, a focus on the energy conservation mechanisms of the methanogens studied here was crucial to accurate reconstructions of metabolism.

### BLAST-based comparison to closely related species reflects similarities within methanogen genera

**Fig. 3:**
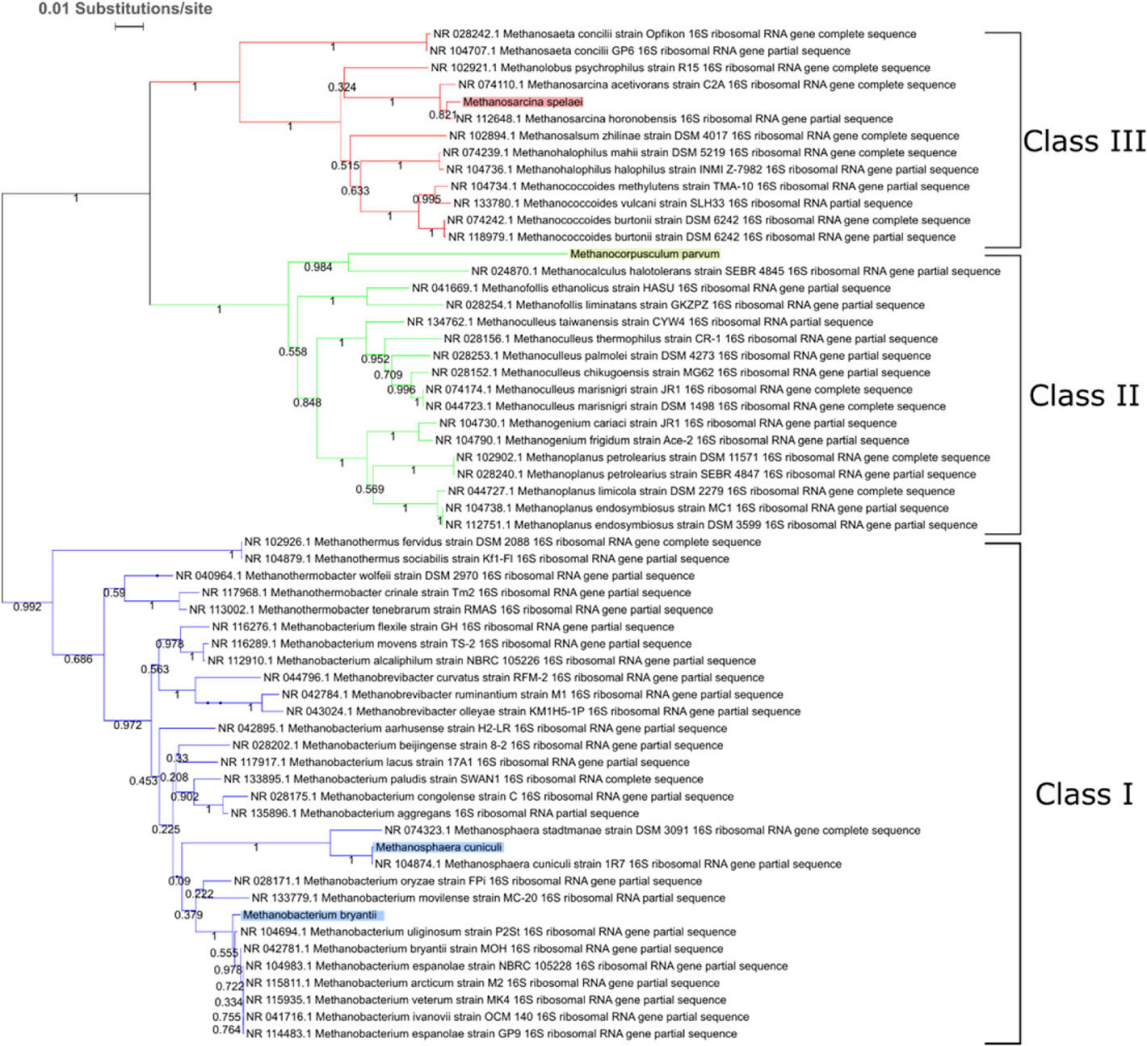
Sequenced methanogens cluster with other methanogens in their respective classes. The phylogenetic tree represents the evolutionary relationship of the sequenced methanogens (highlighted in color by Class) compared to other closely related sequenced archaea. As shown, methanogens in this study displayed a close evolutionary relationship to other methanogens within their genera. Within this tree, the *Methanomicrobiales* (represented by *Mcor. parvum)* are phylogenetically close to the *Methanosarcinales* (*Msar. spelaei)*, but metabolically more similar to the *Methanobacteriales* (*Msph. cuniculi* and *Mbac. bryantii*). Bootstrap values are indicated for each node

The evolutionary relationship of the methanogens was determined by placing them into a phylogenetic tree as shown in Fig. 3. Each methanogen clustered closely with other methanogens from its assigned genus, with the Class I methanogens (*Mbac. bryantii* and *Msph. cuniculi*) distinguished from the Class II methanogen (*Mcor. parvum)* and the Class III methanogen (*Msar. spelaei)*. *Methanosphaera* and *Methanocorpusculum* are both underrepresented in number of species sequenced to date. As such, *Msph. cuniculi* and *Mcor. parvum* were both compared to the other sequenced species in their genera. A BLAST-based comparison was performed to determine the proteins found in *Msph. cuniculi* or *Mcor. parvum* not found in other sequenced species. The results of the BLAST-based comparison are shown in Additional files 2 and 3 for *Msph. cuniculi* and *Mcor. parvum*, respectively. The most striking observation from this comparison is the number of genes annotated as either “hypothetical protein” or with no annotation. Two hundred fifty three of the 277 proteins (91.3%) found in *Msph. cuniculi*, but not in *Methanosphaera stadtmanae,* lacked a descriptive annotation, and the same was true for 230 of the 293 proteins (78.5%) found in *Mcor. parvum,* but not *Methanocorpusculum bavaricum* or *Methanocorpusculum labreanum.* Of the few proteins containing a descriptive annotation, none of them play a significant role in methanogenesis in either isolate. The largest group of proteins annotated in the datasets belonged to either Type I or Type II restriction-modification system, suggesting slight differences in specificity against foreign DNA. Taken together, these observations suggest that *Msph. cuniculi* and *Mcor. parvum* are metabolically similar to other *Methanosphaera* and *Methanocorpusculum*, respectively, and those other sequenced isolates represent good templates for metabolic reconstruction.

### Metabolic reconstruction of methanogenesis pathways

**Fig. 4:**
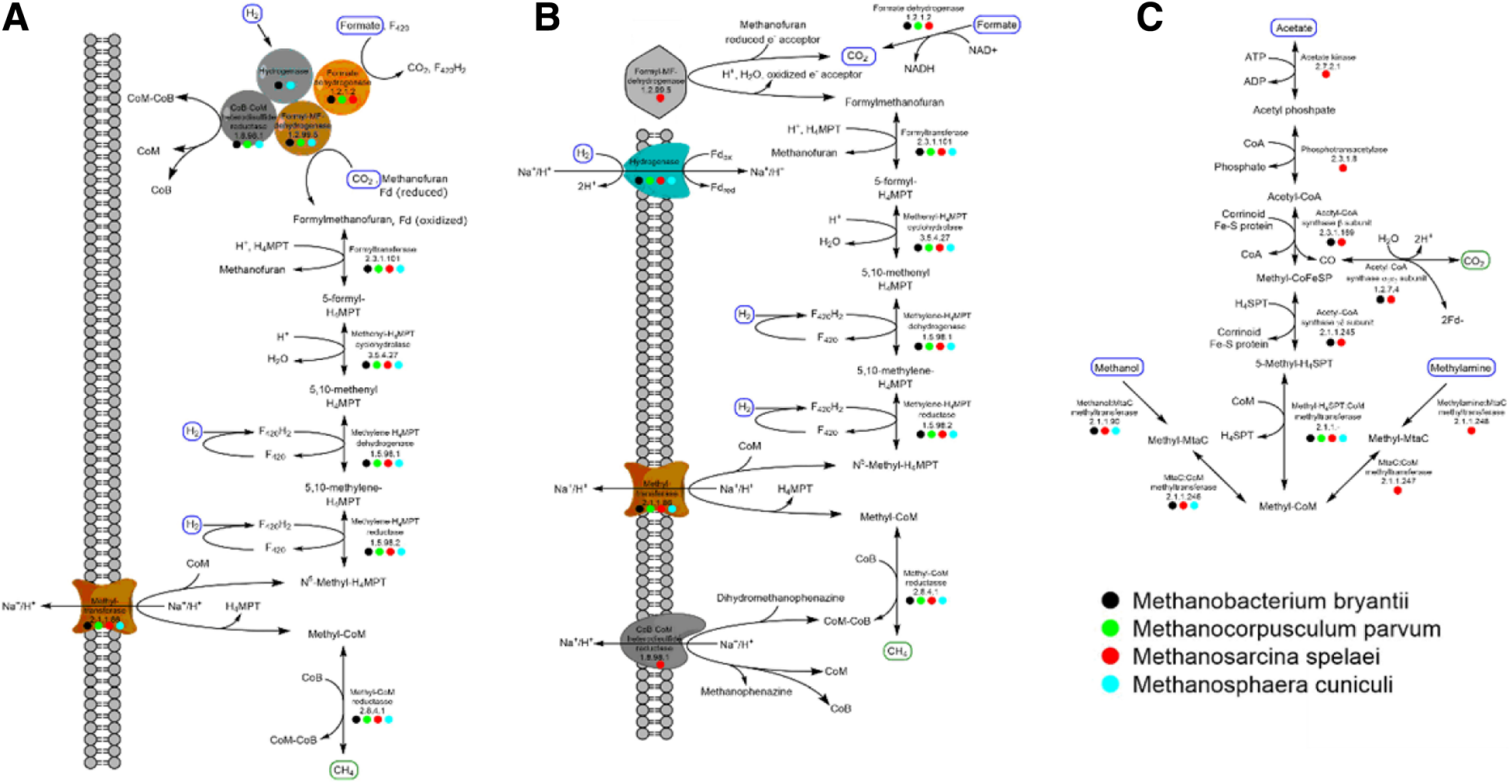
Metabolic reconstruction of methanogenesis reveals two mechanisms of energy conservation. Energy conservation through methanogenesis is detailed for electron bifurcation (**a**) and chemiosmotic coupling (**b**). Electron bifurcation conserves energy in methanogenesis by taking two pairs of electrons from two separate hydrogen molecules and splitting them into a high energy state (CO_2_ or ferredoxin reduction) and a low energy state (CoB-S-S-CoM heterodisulfide reduction). Genes for this mechanism were found in *Msph. cuniculi, Mbac. bryantii,* and *Mcor. parvum*, although the coupled hydrogenase was not identified in *Mcor. parvum*. *Msar. spelaei* utilizes chemiosmotic coupling for energy conservation, where Na^+^ or H^+^ transport into the cell is linked to H_2_ oxidation, and transport out of the cell is linked to methyltransferase and heterodisulfide reductase activity, establishing a net outward gradient for ATP production. The hydrogenase depicted in (**a**) represents the Mvh hydrogenase (*Msph. cuniculi* and *Mbac. bryantii*), and the hydrogenase in (**b**) represents the Eha (*Mbac. bryantii* and *Mcor. parvum*), Ehb (*Mbac. bryantii* and *Msph. cuniculi*), or Ech hydrogenase (*Msar. spelaei* and *Mcor. parvum*). Methylotrophic methanogenesis pathways are displayed in (**c**). Methanol utilization pathways are found in *Msph. cuniculi, Msar. spelaei,* and *Mbac. bryantii*. Acetate and methylamine utilization pathways are found only in *Msar. spelaei*. The acetyl-CoA synthase complex is found in *Mbac. bryantii* and *Msar. spelaei*, allowing them to fix CO_2_

In order to further investigate proteins involved in energy conservation mechanisms, the specific pathways of methanogenesis were reconstructed for each sequenced isolate (Fig. 4). Of particular interest were the distinct energy conservation mechanisms employed by each isolate and the predicted substrate utilization routes. Together, these two aspects provide the backbone for metabolic models, which can be used for predicting behavior in microbial communities.

### *Methanobacterium bryantii*

As a member of the Class I methanogens, *Mbac. bryantii* was expected to utilize a cytoplasmic heterodisulfide reductase complex (Hdr, CDS.4429–30), coupled to a non-F_420_ reducing hydrogenase (Mvh, CDS.4299–302) [53]. These two enzymes couple with the formylmethanofuran dehydrogenase complex (Fwd, CDS.4483–89), which allows for electron bifurcation between the Mvh and Hdr, where two pairs of electrons are split between a thermodynamically unfavorable reaction (CO_2_ reduction to formylmethanofuran or reduction of ferredoxin) and a thermodynamically favorable reaction (heterodisulfide reduction) [51]. As shown in Fig. 4 and Table 3, *Mbac. bryantii* contains these components and likely catalyzes methanogenesis through electron bifurcation. *Mbac. bryantii* has all the genes necessary for its previously reported phenotypic growth on H_2_/CO_2,_ and contains genes for an acetyl-CoA synthase complex (CDS.3915–3920), allowing it to fix CO_2_ for central metabolism. The Fwd complex, which requires either tungsten or molybdenum as a cofactor [54], was found in close proximity to genes related to molybdenum transport (CDS.4477–80), molybdopterin synthesis (CDS.4490), and tungsten transport (CDS.4475), as seen in other methanogens [55]. The clustering of genes suggests that they may be co-regulated to aid in complex formation. Two sets of energy-conserving hydrogenases, Eha (CDS.5584–90) and Ehb (CDS.6156–70), were found in the genome of *Methanobacterium bryantii*. Eha and Ehb have been associated with anaplerosis [56] and CO_2_ assimilation [57], respectively, during hydrogenotrophic methanogenesis in *Methanococcus maripaludis*.

**Table 3:** Hydrogenase Components of the Methanogens in this Study

Species	Hydrogenases	Components
*Mbac. bryantii*	Eha Ehb Mvh Frh	B,C,F,G,H,J,M,N,O,P,P3,R,gene2(V) D,E,F,H,I,K,L,M,N,O,Q A,D,G A,B,G
*Msph. cuniculi*	Ehb Mvh Frh	A,B,C,D,E,F,G,H,I,J,K,L,M,N,O,Q A,D,G A,B,G
*Msar. spelaei*	Ech Cytochrome b Vho Frh	A,B,C,D,E,F I,II A,C,G A,B,G
*Mcor. parvum*	Ech Mbh Eha Ehb Frh	A,B,C,D,E,F A,B,C,D,E,F,G,H,I,J,K,L,M,N B,C,D,E,F,G,H,J,M,N,O Q A,B,G

*Mbac. bryantii* possesses several genes for metabolic substrate utilization not previously observed from *Mbac. bryantii* in isolation. It contains several copies of the formate dehydrogenase genes (CDS.3844–7, CDS.333–4, CDS.6040–4) along with a formate transporter (CDS.6039), suggesting the possibility of growth on formate, although this phenotype in *Mbac. bryantii* has not been reported previously [58]. It also contains the complete set of genes necessary for methanogenesis from methanol/H_2_, including a methyl transferase (CDS.5844), corrinoid protein (CDS.5843), and corrinoid activating protein (CDS.5842)*.* A sulfite reductase gene is also present (CDS.5502), which likely explains the previously reported observation that it resists sulfite inhibition [59]. Finally, *Mbac. bryantii’*s genome possesses an alcohol dehydrogenase (CDS.3886) and NADP-dependent F_420_ reductase (CDS.3694), suggesting the possibility of growth on isopropanol or isobutanol, sometimes seen in other *Methanobacterium* species [46]. However, attempts at growth on formate, methanol, and isopropanol were unsuccessful under conditions tested in this study, suggesting that the genes responsible are inactive or the proper conditions for gene activation were unmet. The presence of the noted genes are important, however, when considering how *Mbac. bryantii* might act in co-culture or in its native environment. For example, both formate and hydrogen are commonly used to transfer electrons in microbial consortia [60], so it is possible that formate metabolism is triggered by low partial pressures of H_2_.

### *Methanosphaera cuniculi*

A genomic analysis of *Msph. cuniculi* revealed that it is metabolically similar to the other *Methanosphaera* sequenced, *Msph. stadtmanae. Msph. stadtmanae* is known to have a restrictive metabolism, requiring both methanol and H_2_ for growth [61]. Like *Msph. stadtmanae, Msph. cuniculi* contains the genes encoding the non-F_420_-reducing hydrogenase, Mvh (CDS.1980–2) [61]. As with *Mbac. bryantii,* this hydrogenase likely couples with the heterodisulfide reductase complex, Hdr (CDS.2562–4) [51]. The heterodisulfide reduction is likely coupled to oxidation of two H_2_ molecules and ferredoxin reduction. The ferredoxin is then either used for anabolic processes or oxidized by Ehb (CDS.2129–49) in an energy-conserving mechanism producing H_2_ and transporting ions out of the cell, as proposed recently by Thauer et al. [5].

The utilization of methanol likely occurs through the action of three proteins: a corrinoid protein (CDS.2507), a methyltransferase corrinoid activation protein (CDS.2506), and a methanol methyltransferase protein (CDS.2508) [62]. These proteins transfer a methyl group from methanol, to the corrinoid protein, and finally to methyl-coenzyme M. Beyond methanol metabolism, *Msph. cuniculi* contains all the genes necessary for reduction of CO_2_ for methanogenesis, as shown in Fig. 4a. However, previous characterization has concluded that it requires methanol for growth [45]. The lack of growth is most likely explained by the requirement of molybdopterin by the formylmethanofuran dehydrogenase, and the lack of enzymes capable of producing molybdopterin in *Msph. cuniculi.* However, it is also possible that genes are not functional in vivo. Like *Mbac. bryantii*, it is important to note the potentially novel substrate routes, as *Msph. cuniculi* may be capable of utilizing CO_2_ and H_2_ in a native environment or in conditions not seen in isolation.

### *Methanosarcina spelaei*

Instead of utilizing electron bifurcation to couple the unfavorable reduction of CO_2_ to the favorable reduction of the CoM-S-S-CoB heterodisulfide, H_2_-utilizing *Methanosarcina* couple each step to ion transport, termed chemiosmotic coupling, shown in Fig. 4b [5]. The favorable step of heterodisulfide reduction is coupled to transport of H^+^ or Na^+^ out of the cell, establishing a gradient [5]. This step is performed by a methanophenazine reducing hydrogenase (Vho, CDS.8117–25) / heterodisulfide reductase (Hdr, CDS.5944–6) complex [5]. Part of this gradient is then used by the energy conserving hydrogenase (Ech, CDS.6169–74) to reduce ferredoxin with H_2_ [5]. Finally, the ferredoxin is utilized to reduce CO_2_ by the formylmethanofuran dehydrogenase complex (Fmd, CDS.9330–4) [5]. As shown in Fig. 4b and Table 3, *Msar. spelaei’s* genome contains all of the genes required for this process. Additionally, the membrane protein and transporter analysis (Additional files 4 and 5) revealed that the Ech and Hdr complexes are predicted to be membrane-bound and catalytically active transporters, which has been experimentally validated in other isolates [63, 64]. The genome for *Msar. spelaei* contains the F_420_-reducing hydrogenase (FrhABG, CDS.9055–8), used for CO_2_ reduction. It also contains a methanophenazine-dependent F_420_H_2_ dehydrogenase complex (Fpo, CDS.7883–95), which reduces methanophenazine with F_420_H_2_ while transporting two protons across the membrane. This allows for the interconversion of reduced cofactors (methanophenazine and F_420_) while conserving energy, which is important for methylotrophic methanogenesis [65].

Genome sequencing of *Msar. spelaei* presents an explanation for its observed methanogenesis on methanol, acetate, and methylamines, as shown in Fig. 4c. Methyltransferases specific to methanol (CDS.8578–80), monomethylamine (CDS.10223–4), dimethylamine (CDS.10167–8), and trimethylamine (CDS.9576,9578) were all identified within the genome. Each methylamine methyltransferase includes an amber stop codon that putatively encodes a pyrrolysine residue, which has been shown to be critical for activity in *Methanosarcina acetivorans* [66]. Supporting the synthesis of this non-canonical amino acid are proline reductase, pyrrolysine synthetase, proline 2-methylase, and pyrrolysyl-tRNA synthetase, all of which were found in the same region of the genome as the monomethylamine methyltransferase complex (CDS.7548–51) [67]. Finally, an acetate kinase (CDS.6562) and phosphotransacetylase (CDS.6561) were found, explaining growth on acetate [68].

### *Methanocorpusculum parvum*

**Fig. 5:**
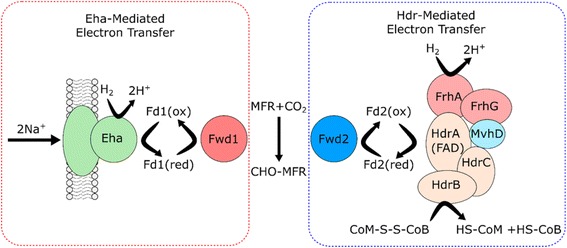
The proposed role for each of the two copies of formylmethanofuran dehydrogenase in *Methanocorpusculum parvum*. *Mcor. parvum* contains two copies of the formylmethanofuran dehydrogenase complex, one clustered with the Eha energy conserving hydrogenase and one clustered with the heterodisulfide reductase (HdrABC/MvhD) complex. We propose that the two complexes are specific to different ferredoxins, helping to separate the electron pool utilized for anabolism from that utilized to replenish methanogenesis intermediates as previously proposed [49]. The Eha-mediated electron transfer results in no net energy gain through methanogenesis, but replenishes intermediates lost to leaky electron bifurcation or biosynthesis and anabolism. The Hdr mediated electron transfer functions similarly to Class 1 methanogens through electron bifurcation, resulting in a net gain of energy from methanogenesis. Since MvhAG are not present, the best explanation is through use of FrhAG, however that association still needs to be experimentally verified.

As shown in Fig. 4, *Mcor. parvum* has all the genes necessary for methanogenesis with H_2_/CO_2_ and formate as substrates. *Mcor. parvum* lacks the acetyl-CoA synthase (Fig. 4c), uncoupling methanogenesis from CO_2_ fixation into acetyl-CoA, which explains its phenotypic requirement for acetate or yeast extract for carbon assimilation [44]. It contains the most diverse set of hydrogenases of the methanogens studied here, as shown in Table 3. Two separate gene clusters were found within the genome containing the formylmethanofuran dehydrogenase complex, Fwd. In one case, the Fwd genes (CDS.2339–40) are clustered with an energy conserving hydrogenase, Eha (CDS.2325–37). Several of the Eha components are predicted to be membrane associated (Additional file 4) with a predicted function of coupling Fwd activity to transmembrane sodium or proton transport according to the transporter analysis (Additional file 5) [5]. A second cluster contains genes for the Fwd complex (CDS.3326–9) along with the heterodisulfide reductase (HdrABC, CDS.3322–4), including two HdrA subunits and one each of the HdrB and HdrC subunits. None of the associated subunits contain predicted transmembrane domains, suggesting that the Hdr complex is cytosolic, as previously determined [5]. Unlike members of the Class I methanogens or the *Methanocellales* species [69], the complementary [NiFe] hydrogenase (MvhAG) was not identified in the *Mcor. parvum* genome, but the MvhD subunit was present. The FrhABG hydrogenase (CDS.2912–15) was identified as well. The presence of two separate gene clusters coding for the Fwd complex suggest that they may be expressed in response to different growth conditions, or play different roles in methanogenesis. The Eha hydrogenase has been shown to be critical for replenishing intermediates of methanogenesis lost to leaky electron bifurcation, dilution due to growth, or biosynthesis [56]. It was also proposed that a separate ferredoxin was utilized in order to separate electron pools for anabolism and replenishment of intermediates [56]. The weak similarity of the two Fwd complexes in *Mcor. parvum* (~36–44% amino acid identity of each component), could represent differences in specificity of the ferredoxin used – one specific for ferredoxin reduced by Eha, and one specific for ferredoxin reduced by a putative electron bifurcating Hdr complex. Separate ferredoxin proteins are encoded in each of the Fwd gene clusters, further supporting this hypothesis. The exact hydrogenase utilized in the Hdr complex has yet to be experimentally determined, however the most likely hypothesis is that the FrhAG components also serve to replace the MvhAG used in other methanogens [54], resulting in a FrhAG/MvhD/HdrABC complex, as shown in Fig. 5. Interestingly, a recent study reported the heterologous expression in *Escherichia coli* of the cytoplasmic HdrA2B2C2 complex from *Methanosarcina acetivorans* [70]. The HdrA subunit was a fusion of the HdrA/MvhD subunits and was capable of directly utilizing F_420_H_2_. While it shares only 42% identity with the concatenated HdrA/MvhD found separately in *Mcor. parvum*¸ it provides biochemical evidence for an alternative model in which the Frh complex generates F_420_H_2_ separately to be utilized by the MvhD/HdrABC complex, thus alleviating the need for MvhAG subunits.

Genes for formate dehydrogenase were found in *Mcor. parvum* (CDS.1907–8, CDS.2816–17), but were located outside of the Fwd/Hdr cluster. Formate dehydrogenase has been shown to form a complex with the Hdr enzyme complex, allowing the electrons to flow directly from formate to the heterodisulfide bond [71]. This observation links directly to the observed phenotype of growth on formate by *Mcor. parvum.* Finally, this cluster also contains a gene annotated as either a sulfite reductase or nitrite reductase (CDS.3330). These enzymes have previously been reported in methanogens, but never specifically clustered with methanogenesis genes [72]. Similar clusters are seen in sulfate reducing organisms, where a sulfite reductase is coupled to an Hdr-like complex [73]. The sulfite reductase located in close proximity to the Hdr complex suggests that it may have been acquired at the same time as the Hdr genes, or perhaps that the protein closely associates with the Fdh/Hdr complex in order to accept electrons from formate.

In addition to H_2_/CO_2_ and formate, *Mcor. parvum* is capable of growth and methanogenesis from isopropanol [74]. The genome of *Mcor. parvum* lacked proteins annotated as alcohol dehydrogenases. Previously, an alcohol dehydrogenase was found in *Mcor. parvum,* and the protein’s N-terminus was sequenced [75]. By searching the genome for this N-terminal sequence, a gene (CDS.2878) annotated as a threonine dehydrogenase was the only match to this sequence. The threonine dehydrogenase aligned with other alcohol dehydrogenases with >70% identity, suggesting that this gene likely functions as the NADP-dependent alcohol dehydrogenase, converting 2-propanol and NADP to acetone and NADPH, respectively.

## Conclusions

The diverse group of methanogens sequenced in this study and the careful reconstruction of their metabolisms have brought forth several new hypotheses as to how methanogens may behave in communities compared to how they behave in isolation. The genome of *Msph. cuniculi* represents only the 2nd sequenced genome from *Methanosphaera,* and the genome of *Mcor. parvum* represents the 3rd from *Methanocorpusculum,* so their genomes fill in gaps of underrepresented genera*.* Collectively, methanogens occupy a unique ecological niche, acting as the terminal electron acceptors in anaerobic environments. As demonstrated by the COG analysis, significantly more proteins are used for energy conservation and for coenzyme metabolism than a representative sample from Genbank, enabling the unique metabolism of the methanogens. Many of the hydrogenases and other proteins require coenzymes and ion cofactors, which require protein resources to attain. The vast majority of membrane proteins belong to the general classes of catalysis and energy conservation, ion and metabolite transport, and metal and cofactor acquisition. Methanogens have evolved to occupy the niche of utilizing CO_2_ or acetate as the primary terminal electron acceptor [76], and their genomes reflect this adaptation.

Energy conservation is predicted to occur through chemiosmotic coupling in *Msar. spelaei* and through electron bifurcation linked to chemiosmotic coupling in *Mbac. bryantii* and *Msph. cuniculi*. *Mcor. parvum*, however, lacks the cytoplasmic non-F_420_ reducing hydrogenase typically utilized for electron bifurcation and lacks the membrane-associated heterodisulfide reductase utilized for chemiosmotic coupling. The lack of a membrane-associated Hdr complex in *Mcor. parvum* suggests that electron bifurcation must take place, with the most likely explanation being that the FrhAG subunits replace the MvhAG found in Class I methanogens. The exact mechanism for conservation in *Mcor. parvum* and other members of the *Methanomicrobiales*, however, needs to be determined biochemically.

Metabolic analysis, supported by previous biochemical research, has led to new predicted growth phenotypes in the sequenced methanogens. We have assembled draft metabolic models for the four isolates, which serve as the starting point for construction of models of other methanogens, or further studies to refine the metabolic map of these strains with techniques like Flux Balance Analysis. While none of the newly predicted growth phenotypes were demonstrated in isolation here, their presence is important to understanding how methanogens coexist in microbial communities. For example, *Mbac. bryantii*’s genome contained the most predicted new phenotypes, and it is a known syntrophic organism. Its activity in a native community could yield phenotypes that are different from those displayed in isolation. Several studies have demonstrated the effect of pH_2_ on gene expression in methanogens [77, 78]. It is possible that the low H_2_ threshold exhibited by *Mbac. bryantii* [79] triggers expression of genes for this phenotype, and that this constant level of low *P*_H2_ can only be achieved in co-culture. Additionally, direct electron transfer has been demonstrated for methanogen-containing co-cultures [80], which could explain the presence of the formylmethanofuran dehydrogenase in *Msph. cuniculi*. Similarly, *Methanosaeta harundinacea* was unable to reduce CO_2_ in isolation, but through the action of direct electron transfer, it could utilize the formylmethanofuran dehydrogenase in its genome [80].

## Additional files

Additional file 1:  Supplementary Information. (DOCX 1394 kb)

Additional file 2:  Genes in *Msph. cuniculi *not in other *Methanosphaera*. (XLSX 13 kb)

Additional file 3:  Genes in *Mcor. parvum *not in other *Methanocorpusculum*. (XLSX 16 kb)

Additional file 4:  Predicted Membrane Proteins. (XLSX 206 kb)

Additional file 5:  Predicted Transporter Proteins. (XLSX 39 kb)

## Abbreviations

COG: Clusters of Orthologous Groups
EC: Enzyme Commission
Fpo: Methanophenazine-dependent F420H2 dehydrogenase complex
Fwd: Formylmethanofuran dehydrogenase
Hdr: Heterodisulfide reductase complex
KEGG: Kyoto Encyclopedia of Genes and Genomes
Mbac: Methanobacterium
Mcor: Methanocorpusculum
Msar: Methanosarcina
Msph: Methanosphaera
Mvh: Non-F420 reducing hydrogenase
TCDB: Transporter Classification DataBase
TMHMM: Transmembrane Hidden Markov Model
Vho: Methanophenazine reducing hydrogenase

## References

[1] Liu Y, Whitman WB (2008). Metabolic, phylogenetic, and ecological diversity of the methanogenic archaea. Ann N Y Acad Sci.

[2] Bang C, Schmitz RA (2015). Archaea associated with human surfaces: not to be underestimated. FEMS Microbiol Rev.

[3] Costa KC, Leigh JA (2014). Metabolic versatility in methanogens. Curr Opin Biotechnol.

[4] Klüpfel L, Piepenbrock A, Kappler A, Sander M (2014). Humic substances as fully regenerable electron acceptors in recurrently anoxic environments. Nat Geosci.

[5] Thauer RK, Kaster A-K, Seedorf H, Buckel W, Hedderich R (2008). Methanogenic archaea: ecologically relevant differences in energy conservation. Nat Rev Microbiol.

[6] Catlett JL, Ortiz AM, Buan NR (2015). Rerouting cellular electron flux to increase the rate of biological methane production. Appl Environ Microbiol.

[7] Soo VW, McAnulty MJ, Tripathi A, Zhu F, Zhang L, Hatzakis E, Smith PB, Agrawal S, Nazem-Bokaee H, Gopalakrishnan S (2016). Reversing methanogenesis to capture methane for liquid biofuel precursors. Microb Cell Factories.

[8] Scheehle EA, Kruger D (2006). Global anthropogenic methane and nitrous oxide emissions. Energy J.

[9] Leahy SC, Kelly WJ, Altermann E, Ronimus RS, Yeoman CJ, Pacheco DM, Li D, Kong Z, McTavish S, Sang C (2010). The genome sequence of the rumen methanogen *Methanobrevibacter ruminantium* reveals new possibilities for controlling ruminant methane emissions. PLoS One.

[10] Lyu Z, Jain R, Smith P, Fetchko T, Yan Y, Whitman WB (2016). Engineering the autotroph *Methanococcus maripaludis* for geraniol production. ACS Synth Biol.

[11] Demirel B, Schere P (2008). The roles of acetotrophic and hydrogenotrophic methanogens during anaerobic conversion of biomass to methane: a review. Rev Environ Sci Biotechnol.

[12] Solomon KV, Haitjema CH, Henske JK, Gilmore SP, Borges-Rivera D, Lipzen A, Brewer HM, Purvine SO, Wright AT, Theodorou MK (2016). Early-branching gut fungi possess a large, comprehensive array of biomass-degrading enzymes. Science.

[13] Peng X, Gilmore S, O'Malley M (2016). Microbial communities for bioprocessing: lessons learned from nature. Current Opinion in Chemical Engineering.

[14] Haitjema CH, Gilmore SP, Henske JK, Solomon KV, de Groot R, Kuo A, Mondo SJ, Salamov AA, LaButti K, Zhao Z (2017). A parts list for fungal cellulosomes revealed by comparative genomics. Nature Microbiology.

[15] Samuel BS, Hansen EE, Manchester JK, Coutinho PM, Henrissat B, Fulton R, Latreille P, Kim K, Wilson RK, Gordon JI (2007). Genomic and metabolic adaptations of *Methanobrevibacter smithii* to the human gut. Proc Natl Acad Sci.

[16] Bapteste E, Brochier C, Boucher Y (2005). Higher-level classification of the Archaea: evolution of methanogenesis and methanogens. Archaea.

[17] Anderson IJ, Sieprawska-Lupa M, Goltsman E, Lapidus A, Copeland A, Del Rio TG, Tice H, Dalin E, Barry K, Pitluck S (2009). Complete genome sequence of *Methanocorpusculum labreanum* type strain Z. Stand Genomic Sci.

[18] Anderson I, Ulrich LE, Lupa B, Susanti D, Porat I, Hooper SD, Lykidis A, Sieprawska-Lupa M, Dharmarajan L, Goltsman E (2009). Genomic characterization of methanomicrobiales reveals three classes of methanogens. PLoS One.

[19] Borrel G, Parisot N, Harris HM, Peyretaillade E, Gaci N, Tottey W, Bardot O, Raymann K, Gribaldo S, Peyret P (2014). Comparative genomics highlights the unique biology of *Methanomassiliicoccales*, a Thermoplasmatales-related seventh order of methanogenic archaea that encodes pyrrolysine. BMC Genomics.

[20] Sorokin DY, Makarova KS, Abbas B, Ferrer M, Golyshin PN, Galinski EA, Ciordia S, Mena MC, Merkel AY, Wolf YI, et al. Discovery of extremely halophilic, methyl-reducing euryarchaea provides insights into the evolutionary origin of methanogenesis. Nature Microbiology. 2017;2 10.1038/nmicrobiol.2017.81 PMC5494993 28555626

[21] Teunissen MJ, Dencamp H, Orpin CG, Veld J, Vogels GD (1991). Comparison of growth characteristics of anaerobic fungi isolated from ruminant and non-ruminant herbivores during cultivation in a defined medium. J Gen Microbiol.

[22] Lowe SE, Theodorou MK, Trinci APJ, Hespell RB (1985). Growth of anaerobic rumen fungi on defined and semi-defined media lacking rumen fluid. J Gen Microbiol.

[23] Nikolenko SI, Korobeynikov AI, Alekseyev MA (2013). BayesHammer: Bayesian clustering for error correction in single-cell sequencing. BMC Genomics.

[24] Bankevich A, Nurk S, Antipov D, Gurevich AA, Dvorkin M, Kulikov AS, Lesin VM, Nikolenko SI, Son P, Prjibelski AD (2012). SPAdes: a new genome assembly algorithm and its applications to single-cell sequencing. J Comput Biol.

[25] Zerbino DR, Birney E (2008). Velvet: algorithms for *de novo* short read assembly using de Bruijn graphs. Genome Res.

[26] Brettin T, Davis JJ, Disz T, Edwards RA, Gerdes S, Olsen GJ, Olson R, Overbeek R, Parrello B, Pusch GD (2015). RASTtk: a modular and extensible implementation of the RAST algorithm for building custom annotation pipelines and annotating batches of genomes. Sci Rep.

[27] Kanehisa M, Goto S, Sato Y, Furumichi M, Tanabe M (2012). KEGG for integration and interpretation of large-scale molecular data sets. Nucleic Acids Res.

[28] Boetzer M, Henkel CV, Jansen HJ, Butler D, Pirovano W (2011). Scaffolding pre-assembled contigs using SSPACE. Bioinformatics.

[29] Marchler-Bauer A, Derbyshire MK, Gonzales NR, Lu S, Chitsaz F, Geer LY, Geer RC, He J, Gwadz M, Hurwitz DI (2015). CDD: NCBI's conserved domain database. Nucleic Acids Res.

[30] Alikhan NF, Petty NK, Ben Zakour NL, Beatson SA (2011). BLAST ring image generator (BRIG): simple prokaryote genome comparisons. BMC Genomics.

[31] Stothard P, Wishart DS (2005). Circular genome visualization and exploration using CGView. Bioinformatics.

[32] Tatusova T, Ciufo S, Fedorov B, O'Neill K, Tolstoy I (2014). RefSeq microbial genomes database: new representation and annotation strategy. Nucleic Acids Res.

[33] Rodriguez-Brito B, Rohwer F, Edwards RA (2006). An application of statistics to comparative metagenomics. BMC Bioinformatics.

[34] Saitou N, Nei M (1987). The neighbor-joining method: a new method for reconstructing phylogenetic trees. Mol Biol Evol.

[35] Felenstein J (1985). Confidence limits on phylogenies: an approach using the bootstrap on JSTOR. Evolution.

[36] Tamura K, Nei M, Kumar S (2004). Prospects for inferring very large phylogenies by using the neighbor-joining method. Proc Natl Acad Sci.

[37] Kumar S, Stecher G, Tamura K (2016). MEGA7: molecular evolutionary genetics analysis version 7.0 for bigger datasets. Mol Biol Evol.

[38] Letunic I, Bork P (2016). Interactive tree of life (iTOL) v3: an online tool for the display and annotation of phylogenetic and other trees. Nucleic Acids Res.

[39] Caspi R, Altman T, Dreher K, Fulcher CA, Subhraveti P, Keseler IM, Kothari A, Krummenacker M, Latendresse M, Mueller LA (2012). The MetaCyc database of metabolic pathways and enzymes and the BioCyc collection of pathway/genome databases. Nucleic Acids Res.

[40] Blaut M (1994). Metabolism of methanogens. Antonie Van Leeuwenhoek.

[41] Bairoch A (2000). The ENZYME database in 2000. Nucleic Acids Res.

[42] Krogh A, Larsson B, von Heijne G, Sonnhammer EL (2001). Predicting transmembrane protein topology with a hidden Markov model: application to complete genomes. J Mol Biol.

[43] Saier MH, Tran CV, Barabote RD (2006). TCDB: the transporter classification database for membrane transport protein analyses and information. Nucleic Acids Res.

[44] Zellner G, Alten C, Stackebrandt E, Demacario EC, Winter J (1987). Isolation and characterization of *Methanocorpusculum parvum*, gen-nov, spec-nov, a new tungsten requiring, coccoid methanogen. Arch Microbiol.

[45] Biavati B, Vasta M, Ferry JG (1988). Isolation and characterization of *Methanosphaera cuniculi* sp. nov. Appl Environ Microbiol.

[46] Cadillo-Quiroz H, Brauer SL, Goodson N, Yavitt JB, Zinder SH (2014). Methanobacterium paludis sp. nov. and a novel strain of Methanobacterium lacus isolated from northern peatlands. Int J Syst Evol Microbiol.

[47] Bryant MP, Wolin EA, Wolin MJ, Wolfe RS (1967). *Methanobacillus omelianskii*, a symbiotic association of two species of bacteria. Arch Mikrobiol.

[48] Ganzert L, Schirmack J, Alawi M, Mangelsdorf K, Sand W, Hillebrand-Voiculescu A, Wagner D (2014). *Methanosarcina spelaei* sp nov., a methanogenic archaeon isolated from a floating biofilm of a subsurface sulphurous lake. Int J Syst Evol Microbiol.

[49] Povolotskaya IS, Kondrashov FA, Ledda A, Vlasov PK (2012). Stop codons in bacteria are not selectively equivalent. Biol Direct.

[50] Deppenmeier U (2002). The unique biochemistry of methanogenesis. Prog Nucleic Acid Res Mol Biol.

[51] Kaster AK, Moll J, Parey K, Thauer RK (2011). Coupling of ferredoxin and heterodisulfide reduction via electron bifurcation in hydrogenotrophic methanogenic archaea. Proc Natl Acad Sci.

[52] Benedict MN, Gonnerman MC, Metcalf WW, Price ND (2012). Genome-scale metabolic reconstruction and hypothesis testing in the methanogenic archaeon *Methanosarcina acetivorans* C2A. J Bacteriol.

[53] Setzke E, Hedderich R, Heiden S, Thauer RK (1994). H2: heterodisulfide oxidoreductase complex from *Methanobacterium thermoautotrophicum*. Composition and properties. Eur J Biochem.

[54] Thauer RK, Kaster AK, Goenrich M, Schick M, Hiromoto T, Shima S (2010). Hydrogenases from methanogenic archaea, nickel, a novel cofactor, and H2 storage. Annu Rev Biochem.

[55] Kaster AK, Goenrich M, Seedorf H, Liesegang H, Wollherr A, Gottschalk G, Thauer RK (2011). More than 200 genes required for methane formation from H(2) and CO(2) and energy conservation are present in *Methanothermobacter marburgensis* and *Methanothermobacter thermautotrophicus*. Archaea.

[56] Lie TJ, Costa KC, Lupa B, Korpole S, Whitman WB, Leigh JA (2012). Essential anaplerotic role for the energy-converting hydrogenase Eha in hydrogenotrophic methanogenesis. Proc Natl Acad Sci.

[57] Porat I, Kim W, Hendrickson EL, Xia Q, Zhang Y, Wang T, Taub F, Moore BC, Anderson IJ, Hackett M (2006). Disruption of the operon encoding Ehb hydrogenase limits anabolic CO2 assimilation in the archaeon *Methanococcus maripaludis*. J Bacteriol.

[58] Guyot JP, Brauman A (1986). Methane production from formate by syntrophic association of *Methanobacterium bryantii* and *Desulfovibrio vulgaris* JJ. Appl Environ Microbiol.

[59] Kluber HD, Conrad R (1998). Inhibitory effects of nitrate, nitrite, NO and N2O on methanogenesis by *Methanosarcina barkeri* and *Methanobacterium bryantii*. FEMS Microbiol Ecol.

[60] Embree M, Liu JK, Al-Bassam MM, Zengler K (2015). Networks of energetic and metabolic interactions define dynamics in microbial communities. Proc Natl Acad Sci.

[61] Fricke WF, Seedorf H, Henne A, Kruer M, Liesegang H, Hedderich R, Gottschalk G, Thauer RK (2006). The genome sequence of *Methanosphaera stadtmanae* reveals why this human intestinal archaeon is restricted to methanol and H2 for methane formation and ATP synthesis. J Bacteriol.

[62] Sauer K, Harms U, Thauer RK (1997). Methanol:coenzyme M methyltransferase from *Methanosarcina barkeri*. Purification, properties and encoding genes of the corrinoid protein MT1. Eur J Biochem.

[63] Ide T, Bäumer S, Deppenmeier U (1999). Energy conservation by the H2:heterodisulfide oxidoreductase from *Methanosarcina mazei* gö1: identification of two proton-translocating segments. J Bacteriol.

[64] Welte C, Kratzer C, Deppenmeier U (2010). Involvement of Ech hydrogenase in energy conservation of *Methanosarcina mazei*. FEBS J.

[65] Baumer S, Ide T, Jacobi C, Johann A, Gottschalk G, Deppenmeier U (2000). The F420H2 dehydrogenase from *Methanosarcina mazei* is a redox-driven proton pump closely related to NADH dehydrogenases. J Biol Chem.

[66] Mahapatra A, Patel A, Soares JA, Larue RC, Zhang JK, Metcalf WW, Krzycki JA (2006). Characterization of a *Methanosarcina acetivorans* mutant unable to translate UAG as pyrrolysine. Mol Microbiol.

[67] Gaston MA, Zhang L, Green-Church KB, Krzycki JA (2011). The complete biosynthesis of the genetically encoded amino acid pyrrolysine from lysine. Nature.

[68] Ferry JG (1997). Enzymology of the fermentation of acetate to methane by *Methanosarcina thermophila*. Biofactors.

[69] Lyu Z, Lu Y (2015). Comparative genomics of three *Methanocellales* strains reveal novel taxonomic and metabolic features. Environ Microbiol Rep.

[70] Yan Z, Wang M, Ferry JG. A ferredoxin- and F420H2-dependent, electron-bifurcating, heterodisulfide reductase with homologs in the domains bacteria and archaea. MBio. 2017;8 10.1128/mBio.02285-16 PMC5296606 28174314

[71] Costa KC, Wong PM, Wang T, Lie TJ, Dodsworth JA, Swanson I, Burn JA, Hackett M, Leigh JA (2010). Protein complexing in a methanogen suggests electron bifurcation and electron delivery from formate to heterodisulfide reductase. Proc Natl Acad Sci.

[72] Johnson EF, Mukhopadhyay B (2005). A new type of sulfite reductase, a novel coenzyme F420-dependent enzyme, from the methanarchaeon *Methanocaldococcus jannaschii*. J Biol Chem.

[73] Cardoso Pereira IA, Ramos AR, Grein F, Marques MC, da Silva SM, Venceslau SS (2011). A comparative genomic analysis of energy metabolism in sulfate reducing bacteria and archaea. Front Microbiol.

[74] Zellner G, Stackebrandt E, Messner P, Tindall BJ, Demacario EC, Kneifel H, Sleytr UB, Winter J (1989). *Methanocorpusculaceae* fam-nov, represented by *Methanocorpusculum parvum, Methanocorpusculum sinense* spec-nov and *Methanocorpusculum bavaricum* spec-nov. Arch Microbiol.

[75] Bleicher K, Winter J (1991). Purification and properties of F420-dependent and NADP+−dependent alcohol dehydrogenases of *Methanogenium liminatans* and *Methanobacterium palustre*, specific for secondary alcohols. Eur J Biochem.

[76] Valentine DL (2007). Adaptations to energy stress dictate the ecology and evolution of the Archaea. Nat Rev Microbiol.

[77] Hendrickson EL, Haydock AK, Moore BC, Whitman WB, Leigh JA (2007). Functionally distinct genes regulated by hydrogen limitation and growth rate in methanogenic Archaea. Proc Natl Acad Sci.

[78] Mukhopadhyay B, Johnson EF, Wolfe RS (2000). A novel pH2 control on the expression of flagella in the hyperthermophilic strictly hydrogenotrophic methanarchaeaon *Methanococcus jannaschii*. Proc Natl Acad Sci U S A.

[79] Conrad R, Wetter B (1990). Influence of temperature on energetics of hydrogen metabolism in homoacetogenic, methanogenic, and other anaerobic bacteria. Arch Microbiol.

[80] Rotaru A-E, Shrestha PM, Liu F, Shrestha M, Shrestha D, Embree M, Zengler K, Wardman C, Nevin KP, Lovley DR (2014). A new model for electron flow during anaerobic digestion: direct interspecies electron transfer to *Methanosaeta* for the reduction of carbon dioxide to methane. Energy Environ Sci.

