# Supplementary material for: Genomic analysis of methanogenic archaea reveals a shift towards energy conservation
Source: BMC Genomics. 2017 Aug 21;18:639. doi: 10.1186/s12864-017-4036-4 (PMC5563889; doi:10.1186/s12864-017-4036-4)
Supplement: Supplementary file 1 — Supplementary Information. (DOCX 1394 kb) [file 12864_2017_4036_MOESM1_ESM.docx]

**Additional file**

*Figure S1: Analysis of Membrane Protein and Transporter Functions*

*Figure S2: Distribution of COG proteins vs. Number of Genomes Sampled*


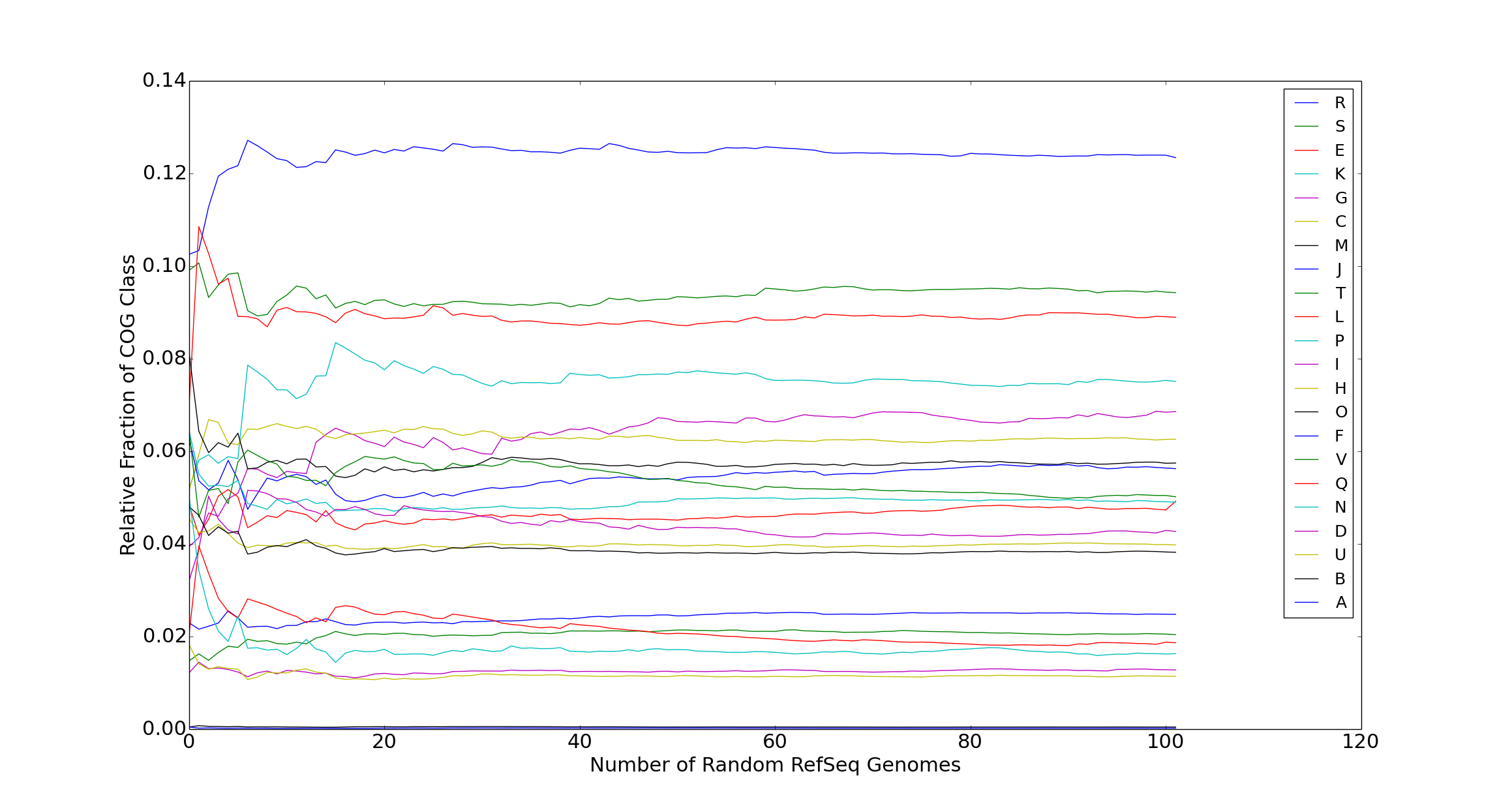


*Figure S3: Bootstrap Analysis of the Sampled RefSeq Genomes*


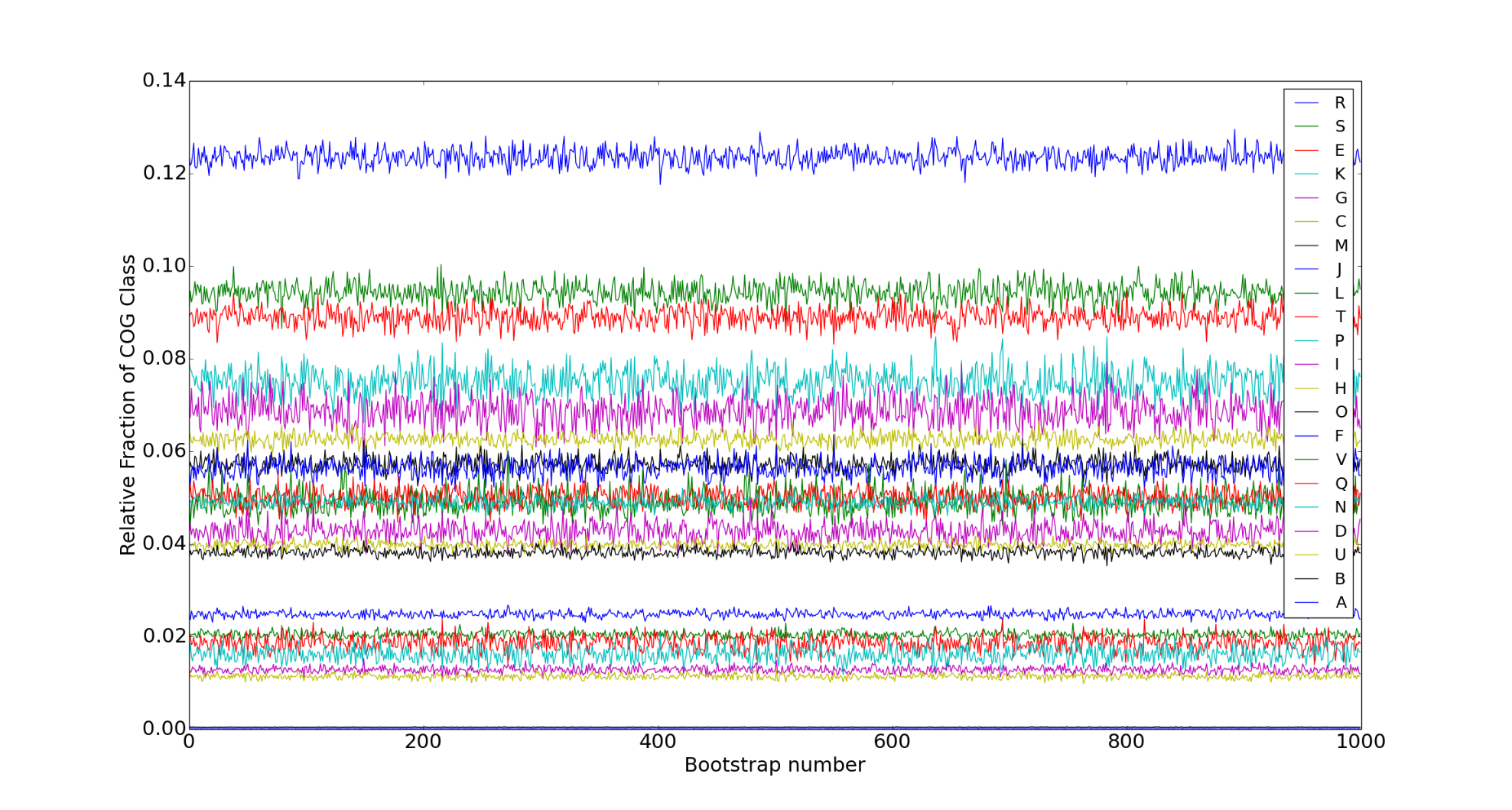


*Table S1: Codon Frequencies*

|  | M. bryantii | M. cuniculi | M. parvum | M. spelaei |
| --- | --- | --- | --- | --- |
| AAA | 0.064387 | 0.072511 | 0.034671 | 0.049774 |
| AAC | 0.017358 | 0.022708 | 0.01827 | 0.019511 |
| AAG | 0.015003 | 0.007108 | 0.020244 | 0.022805 |
| AAT | 0.039024 | 0.052006 | 0.015158 | 0.02893 |
| ACA | 0.022216 | 0.052355 | 0.009915 | 0.017859 |
| ACC | 0.009072 | 0.004251 | 0.024492 | 0.012126 |
| ACG | 0.002774 | 0.000664 | 0.017693 | 0.006386 |
| ACT | 0.019379 | 0.012226 | 0.005913 | 0.017628 |
| AGA | 0.015338 | 0.014496 | 0.00935 | 0.015369 |
| AGC | 0.008413 | 0.003082 | 0.008333 | 0.010359 |
| AGG | 0.009154 | 0.000768 | 0.005248 | 0.011402 |
| AGT | 0.013731 | 0.017425 | 0.005951 | 0.013287 |
| ATA | 0.039935 | 0.059745 | 0.008805 | 0.024397 |
| ATC | 0.013071 | 0.0096 | 0.052953 | 0.019439 |
| ATG | 0.025613 | 0.023271 | 0.028128 | 0.023033 |
| ATT | 0.04037 | 0.033124 | 0.012069 | 0.034237 |
| CAA | 0.011848 | 0.02819 | 0.003319 | 0.009617 |
| CAC | 0.005164 | 0.004856 | 0.008776 | 0.006223 |
| CAG | 0.011742 | 0.003462 | 0.021884 | 0.017044 |
| CAT | 0.01132 | 0.012277 | 0.010006 | 0.010345 |
| CCA | 0.013433 | 0.021896 | 0.004445 | 0.008857 |
| CCC | 0.004164 | 0.000588 | 0.010095 | 0.006198 |
| CCG | 0.002643 | 0.000421 | 0.023093 | 0.006592 |
| CCT | 0.014503 | 0.009763 | 0.007022 | 0.016686 |
| CGA | 0.002628 | 0.004243 | 0.002939 | 0.003718 |
| CGC | 0.001445 | 0.000628 | 0.010163 | 0.003787 |
| CGG | 0.001652 | 0.00041 | 0.013372 | 0.003612 |
| CGT | 0.004118 | 0.009669 | 0.008358 | 0.005071 |
| CTA | 0.00707 | 0.024066 | 0.001398 | 0.006957 |
| CTC | 0.006168 | 0.003669 | 0.026908 | 0.013158 |
| CTG | 0.010108 | 0.001072 | 0.028374 | 0.018894 |
| CTT | 0.024177 | 0.025384 | 0.026114 | 0.032824 |
| GAA | 0.058273 | 0.063488 | 0.034059 | 0.052537 |
| GAC | 0.013265 | 0.009839 | 0.02364 | 0.018866 |
| GAG | 0.011692 | 0.004484 | 0.032256 | 0.022102 |
| GAT | 0.041186 | 0.053552 | 0.0305 | 0.031393 |
| GCA | 0.030859 | 0.039993 | 0.027291 | 0.025826 |
| GCC | 0.007783 | 0.000736 | 0.026484 | 0.012997 |
| GCG | 0.004085 | 0.000305 | 0.022181 | 0.005471 |
| GCT | 0.019794 | 0.012546 | 0.011994 | 0.020163 |
| GGA | 0.029184 | 0.038775 | 0.023791 | 0.028594 |
| GGC | 0.009229 | 0.001102 | 0.024608 | 0.012165 |
| GGG | 0.009574 | 0.001155 | 0.015581 | 0.011513 |
| GGT | 0.020741 | 0.017087 | 0.014156 | 0.014946 |
| GTA | 0.023673 | 0.038526 | 0.008317 | 0.020274 |
| GTC | 0.00619 | 0.001024 | 0.030752 | 0.011858 |
| GTG | 0.008831 | 0.002489 | 0.017908 | 0.011006 |
| GTT | 0.028104 | 0.016797 | 0.017228 | 0.023341 |
| TAA | 0.002576 | 0.002286 | 0.00151 | 0.002379 |
| TAC | 0.012473 | 0.012895 | 0.01413 | 0.013681 |
| TAG | 0.000537 | 0.000539 | 0.000329 | 0.000436 |
| TAT | 0.025136 | 0.030062 | 0.018915 | 0.022801 |
| TCA | 0.018396 | 0.028184 | 0.009246 | 0.015346 |
| TCC | 0.006307 | 0.001798 | 0.01602 | 0.011332 |
| TCG | 0.002298 | 0.000184 | 0.013559 | 0.006137 |
| TCT | 0.015653 | 0.007451 | 0.007112 | 0.014854 |
| TGA | 0.000681 | 0.000304 | 0.001746 | 0.00155 |
| TGC | 0.004195 | 0.001199 | 0.008675 | 0.005987 |
| TGG | 0.008663 | 0.005727 | 0.009045 | 0.010003 |
| TGT | 0.007539 | 0.01026 | 0.006451 | 0.00691 |
| TTA | 0.034447 | 0.024844 | 0.004713 | 0.013672 |
| TTC | 0.011752 | 0.010082 | 0.021159 | 0.016483 |
| TTG | 0.008476 | 0.001451 | 0.005307 | 0.010089 |
| TTT | 0.031385 | 0.024901 | 0.017878 | 0.029166 |

*Table S2: COG categories*

|  | COG Category | M bryantii | M parvum | M spelaei | M cuniculi | RefSeq | | RefSeq Bootstrap Average | RefSeq Bootstrap Standard Deviation |
| --- | --- | --- | --- | --- | --- | --- | --- | --- | --- |
| A | RNA processing and modification | 0.0008464 | 0 | 0.0006636 | 0.0008319 | | 0.0001856 | 0.0001856 | 2.05E-05 |
| B | Chromatin structure and dynamics | 0.0038087 | 0.0014134 | 0.0009954 | 0.0083195 | | 0.000414 | 0.0004151 | 3.67E-05 |
| C | Energy production and conversion | 0.1028354 | 0.0946996 | 0.0816191 | 0.0798669 | | 0.0625716 | 0.0625733 | 0.0012178 |
| D | Cell cycle control, cell division, chromosome partitioning | 0.0084638 | 0.0070671 | 0.0086264 | 0.0066556 | | 0.0127541 | 0.0127758 | 0.0006418 |
| E | Amino acid transport and metabolism | 0.0672873 | 0.0840989 | 0.0769741 | 0.0881864 | | 0.0889113 | 0.0889158 | 0.0019471 |
| F | Nucleotide transport and metabolism | 0.0236987 | 0.034629 | 0.0218978 | 0.0399334 | | 0.0247553 | 0.0247771 | 0.0006324 |
| G | Carbohydrate transport and metabolism | 0.0342785 | 0.034629 | 0.0338421 | 0.0274542 | | 0.0685383 | 0.068542 | 0.0030232 |
| H | Coenzyme transport and metabolism | 0.073212 | 0.0784452 | 0.0613802 | 0.0790349 | | 0.0397576 | 0.0397837 | 0.0007253 |
| I | Lipid transport and metabolism | 0.0143885 | 0.0183746 | 0.0126078 | 0.0133111 | | 0.0426517 | 0.0426434 | 0.001892 |
| J | Translation, ribosomal structure, and biogenesis | 0.0647482 | 0.1024735 | 0.0593895 | 0.1189684 | | 0.0562195 | 0.0563237 | 0.0018627 |
| K | Transcription | 0.0660178 | 0.0508834 | 0.052422 | 0.0490849 | | 0.0750545 | 0.0750944 | 0.0029099 |
| L | Replication, recombination, and repair | 0.0347017 | 0.0459364 | 0.0600531 | 0.0574043 | | 0.0491716 | 0.0491066 | 0.0025058 |
| M | Cell wall/membrane/ envelope biogenesis | 0.0524757 | 0.0261484 | 0.0391506 | 0.0582363 | | 0.0574328 | 0.0573239 | 0.0015513 |
| N | Cell motility | 0.0025391 | 0.0035336 | 0.0079628 | 0.0008319 | | 0.016262 | 0.0161866 | 0.0015398 |
| O | Posttranslational modification, protein turnover, chaperones | 0.0300466 | 0.0416961 | 0.0381553 | 0.0357737 | | 0.0381696 | 0.038151 | 0.0008143 |
| P | Inorganic ion transport and metabolism | 0.0495133 | 0.0565371 | 0.0683477 | 0.0382696 | | 0.0490038 | 0.0489921 | 0.0010222 |
| Q | Secondary metabolites biosynthesis, transport, and catabolism | 0.0080406 | 0.0063604 | 0.0129396 | 0.0024958 | | 0.0186209 | 0.0186208 | 0.0015378 |
| R | General function prediction only | 0.1591198 | 0.15053 | 0.1672196 | 0.1497504 | | 0.1233946 | 0.1234512 | 0.0017524 |
| S | Function unknown | 0.1320355 | 0.1236749 | 0.1270737 | 0.1114809 | | 0.0942357 | 0.0943324 | 0.0020198 |
| T | Signal transduction mechanisms | 0.0440118 | 0.0127208 | 0.0288653 | 0.0033278 | | 0.0501458 | 0.0500646 | 0.0017699 |
| U | Intracellular trafficking, secretion, and vesicular transport | 0.0101566 | 0.0120141 | 0.0136032 | 0.0124792 | | 0.0113695 | 0.0113551 | 0.0005305 |
| V | Defense mechanisms | 0.017774 | 0.0141343 | 0.026211 | 0.0183028 | | 0.0203802 | 0.020386 | 0.0006877 |
| Z | Cytoskeleton | 0 | 0 | 0 | 0 | | 0.0001856 | 0.0001856 | 2.05E-05 |
| Total | Total Genes | 2363 | 1415 | 3014 | 1202 | | 280223 |  |  |

***RefSeq Genomes Sampled List***

ftp://ftp.ncbi.nlm.nih.gov/genomes/all/GCF_000382705.1_ASM38270v1/GCF_000382705.1_ASM38270v1_protein.faa.gz

ftp://ftp.ncbi.nlm.nih.gov/genomes/all/GCF_001267865.1_ASM126786v1/GCF_001267865.1_ASM126786v1_protein.faa.gz

ftp://ftp.ncbi.nlm.nih.gov/genomes/all/GCF_000330885.1_ASM33088v1/GCF_000330885.1_ASM33088v1_protein.faa.gz

ftp://ftp.ncbi.nlm.nih.gov/genomes/all/GCF_000195295.1_ASM19529v1/GCF_000195295.1_ASM19529v1_protein.faa.gz

ftp://ftp.ncbi.nlm.nih.gov/genomes/all/GCF_000701905.1_ASM70190v1/GCF_000701905.1_ASM70190v1_protein.faa.gz

ftp://ftp.ncbi.nlm.nih.gov/genomes/all/GCF_000014925.1_ASM1492v1/GCF_000014925.1_ASM1492v1_protein.faa.gz

ftp://ftp.ncbi.nlm.nih.gov/genomes/all/GCF_000829555.1_ASM82955v1/GCF_000829555.1_ASM82955v1_protein.faa.gz

ftp://ftp.ncbi.nlm.nih.gov/genomes/all/GCF_001443605.1_ASM144360v1/GCF_001443605.1_ASM144360v1_protein.faa.gz

ftp://ftp.ncbi.nlm.nih.gov/genomes/all/GCF_000526315.1_ASM52631v1/GCF_000526315.1_ASM52631v1_protein.faa.gz

ftp://ftp.ncbi.nlm.nih.gov/genomes/all/GCF_000317595.1_Sipom9103v1.0/GCF_000317595.1_Sipom9103v1.0_protein.faa.gz

ftp://ftp.ncbi.nlm.nih.gov/genomes/all/GCF_000576595.1_genome/GCF_000576595.1_genome_protein.faa.gz

ftp://ftp.ncbi.nlm.nih.gov/genomes/all/GCF_000711975.1_ASM71197v1/GCF_000711975.1_ASM71197v1_protein.faa.gz

ftp://ftp.ncbi.nlm.nih.gov/genomes/all/GCF_000817245.1_ASM81724v1/GCF_000817245.1_ASM81724v1_protein.faa.gz

ftp://ftp.ncbi.nlm.nih.gov/genomes/all/GCF_000160455.2_ASM16045v2/GCF_000160455.2_ASM16045v2_protein.faa.gz

ftp://ftp.ncbi.nlm.nih.gov/genomes/all/GCF_000024285.1_ASM2428v1/GCF_000024285.1_ASM2428v1_protein.faa.gz

ftp://ftp.ncbi.nlm.nih.gov/genomes/all/GCF_000970675.1_ASM97067v1/GCF_000970675.1_ASM97067v1_protein.faa.gz

ftp://ftp.ncbi.nlm.nih.gov/genomes/all/GCF_000517625.1_ASM51762v1/GCF_000517625.1_ASM51762v1_protein.faa.gz

ftp://ftp.ncbi.nlm.nih.gov/genomes/all/GCF_001046855.1_ASM104685v1/GCF_001046855.1_ASM104685v1_protein.faa.gz

ftp://ftp.ncbi.nlm.nih.gov/genomes/all/GCF_000785515.1_ASM78551v1/GCF_000785515.1_ASM78551v1_protein.faa.gz

ftp://ftp.ncbi.nlm.nih.gov/genomes/all/GCF_000011365.1_ASM1136v1/GCF_000011365.1_ASM1136v1_protein.faa.gz

ftp://ftp.ncbi.nlm.nih.gov/genomes/all/GCF_001267535.1_ASM126753v1/GCF_001267535.1_ASM126753v1_protein.faa.gz

ftp://ftp.ncbi.nlm.nih.gov/genomes/all/GCF_000023745.1_ASM2374v1/GCF_000023745.1_ASM2374v1_protein.faa.gz

ftp://ftp.ncbi.nlm.nih.gov/genomes/all/GCF_000156295.1_ASM15629v1/GCF_000156295.1_ASM15629v1_protein.faa.gz

ftp://ftp.ncbi.nlm.nih.gov/genomes/all/GCF_000428685.1_ASM42868v1/GCF_000428685.1_ASM42868v1_protein.faa.gz

ftp://ftp.ncbi.nlm.nih.gov/genomes/all/GCF_000336755.1_ASM33675v1/GCF_000336755.1_ASM33675v1_protein.faa.gz

ftp://ftp.ncbi.nlm.nih.gov/genomes/all/GCF_001045455.1_ASM104545v1/GCF_001045455.1_ASM104545v1_protein.faa.gz

ftp://ftp.ncbi.nlm.nih.gov/genomes/all/GCF_000471025.2_ASM47102v2/GCF_000471025.2_ASM47102v2_protein.faa.gz

ftp://ftp.ncbi.nlm.nih.gov/genomes/all/GCF_000367805.1_version_1.0/GCF_000367805.1_version_1.0_protein.faa.gz

ftp://ftp.ncbi.nlm.nih.gov/genomes/all/GCF_001373415.1_Paenibacillus_dakarensis_FF9/GCF_001373415.1_Paenibacillus_dakarensis_FF9_protein.faa.gz

ftp://ftp.ncbi.nlm.nih.gov/genomes/all/GCF_000725425.1_ASM72542v1/GCF_000725425.1_ASM72542v1_protein.faa.gz

ftp://ftp.ncbi.nlm.nih.gov/genomes/all/GCF_000190415.1_ASM19041v1/GCF_000190415.1_ASM19041v1_protein.faa.gz

ftp://ftp.ncbi.nlm.nih.gov/genomes/all/GCF_001314225.1_ASM131422v1/GCF_001314225.1_ASM131422v1_protein.faa.gz

ftp://ftp.ncbi.nlm.nih.gov/genomes/all/GCF_000155775.1_ASM15577v1/GCF_000155775.1_ASM15577v1_protein.faa.gz

ftp://ftp.ncbi.nlm.nih.gov/genomes/all/GCF_000284015.1_ASM28401v1/GCF_000284015.1_ASM28401v1_protein.faa.gz

ftp://ftp.ncbi.nlm.nih.gov/genomes/all/GCF_000794385.1_ASM79438v1/GCF_000794385.1_ASM79438v1_protein.faa.gz

ftp://ftp.ncbi.nlm.nih.gov/genomes/all/GCF_000477435.1_ASM47743v1/GCF_000477435.1_ASM47743v1_protein.faa.gz

ftp://ftp.ncbi.nlm.nih.gov/genomes/all/GCF_000297255.1_ASM29725v1/GCF_000297255.1_ASM29725v1_protein.faa.gz

ftp://ftp.ncbi.nlm.nih.gov/genomes/all/GCF_001466305.1_ASM146630v1/GCF_001466305.1_ASM146630v1_protein.faa.gz

ftp://ftp.ncbi.nlm.nih.gov/genomes/all/GCF_000012385.1_ASM1238v1/GCF_000012385.1_ASM1238v1_protein.faa.gz

ftp://ftp.ncbi.nlm.nih.gov/genomes/all/GCF_000372425.1_ASM37242v1/GCF_000372425.1_ASM37242v1_protein.faa.gz

ftp://ftp.ncbi.nlm.nih.gov/genomes/all/GCF_000218625.1_ASM21862v1/GCF_000218625.1_ASM21862v1_protein.faa.gz

ftp://ftp.ncbi.nlm.nih.gov/genomes/all/GCF_000963705.1_Ellito1.0/GCF_000963705.1_Ellito1.0_protein.faa.gz

ftp://ftp.ncbi.nlm.nih.gov/genomes/all/GCF_000023945.1_ASM2394v1/GCF_000023945.1_ASM2394v1_protein.faa.gz

ftp://ftp.ncbi.nlm.nih.gov/genomes/all/GCF_000176115.2_ASM17611v2/GCF_000176115.2_ASM17611v2_protein.faa.gz

ftp://ftp.ncbi.nlm.nih.gov/genomes/all/GCF_001302585.1_ASM130258v1/GCF_001302585.1_ASM130258v1_protein.faa.gz

ftp://ftp.ncbi.nlm.nih.gov/genomes/all/GCF_000195735.1_ASM19573v1/GCF_000195735.1_ASM19573v1_protein.faa.gz

ftp://ftp.ncbi.nlm.nih.gov/genomes/all/GCF_001402915.1_ASM140291v1/GCF_001402915.1_ASM140291v1_protein.faa.gz

ftp://ftp.ncbi.nlm.nih.gov/genomes/all/GCF_000281085.1_ASM28108v1/GCF_000281085.1_ASM28108v1_protein.faa.gz

ftp://ftp.ncbi.nlm.nih.gov/genomes/all/GCF_000648275.1_ASM64827v1/GCF_000648275.1_ASM64827v1_protein.faa.gz

ftp://ftp.ncbi.nlm.nih.gov/genomes/all/GCF_000300995.1_ASM30099v1/GCF_000300995.1_ASM30099v1_protein.faa.gz

ftp://ftp.ncbi.nlm.nih.gov/genomes/all/GCF_001305595.1_ASM130559v1/GCF_001305595.1_ASM130559v1_protein.faa.gz

ftp://ftp.ncbi.nlm.nih.gov/genomes/all/GCF_001083795.1_Nocardiopsis_sp_SBT349_SPAdes_SSPACE/GCF_001083795.1_Nocardiopsis_sp_SBT349_SPAdes_SSPACE_protein.faa.gz

ftp://ftp.ncbi.nlm.nih.gov/genomes/all/GCF_000006925.2_ASM692v2/GCF_000006925.2_ASM692v2_protein.faa.gz

ftp://ftp.ncbi.nlm.nih.gov/genomes/all/GCF_000175575.2_ASM17557v2/GCF_000175575.2_ASM17557v2_protein.faa.gz

ftp://ftp.ncbi.nlm.nih.gov/genomes/all/GCF_000743575.1_ASM74357v1/GCF_000743575.1_ASM74357v1_protein.faa.gz

ftp://ftp.ncbi.nlm.nih.gov/genomes/all/GCF_000975095.1_ASM97509v1/GCF_000975095.1_ASM97509v1_protein.faa.gz

ftp://ftp.ncbi.nlm.nih.gov/genomes/all/GCF_000615445.1_ASM61544v1/GCF_000615445.1_ASM61544v1_protein.faa.gz

ftp://ftp.ncbi.nlm.nih.gov/genomes/all/GCF_001425865.1_Root404/GCF_001425865.1_Root404_protein.faa.gz

ftp://ftp.ncbi.nlm.nih.gov/genomes/all/GCF_001418285.1_ASM141828v1/GCF_001418285.1_ASM141828v1_protein.faa.gz

ftp://ftp.ncbi.nlm.nih.gov/genomes/all/GCF_000485845.1_RicGra1.0/GCF_000485845.1_RicGra1.0_protein.faa.gz

ftp://ftp.ncbi.nlm.nih.gov/genomes/all/GCF_000252445.1_ASM25244v1/GCF_000252445.1_ASM25244v1_protein.faa.gz

ftp://ftp.ncbi.nlm.nih.gov/genomes/all/GCF_000170775.1_ASM17077v1/GCF_000170775.1_ASM17077v1_protein.faa.gz

ftp://ftp.ncbi.nlm.nih.gov/genomes/all/GCF_000368025.1_Acin_parv_CIP108168_V1/GCF_000368025.1_Acin_parv_CIP108168_V1_protein.faa.gz

ftp://ftp.ncbi.nlm.nih.gov/genomes/all/GCF_000423045.1_ASM42304v1/GCF_000423045.1_ASM42304v1_protein.faa.gz

ftp://ftp.ncbi.nlm.nih.gov/genomes/all/GCF_000283015.1_P735.v1.0/GCF_000283015.1_P735.v1.0_protein.faa.gz

ftp://ftp.ncbi.nlm.nih.gov/genomes/all/GCF_001435835.1_ASM143583v1/GCF_001435835.1_ASM143583v1_protein.faa.gz

ftp://ftp.ncbi.nlm.nih.gov/genomes/all/GCF_000716675.1_ASM71667v1/GCF_000716675.1_ASM71667v1_protein.faa.gz

ftp://ftp.ncbi.nlm.nih.gov/genomes/all/GCF_001029455.1_ASM102945v1/GCF_001029455.1_ASM102945v1_protein.faa.gz

ftp://ftp.ncbi.nlm.nih.gov/genomes/all/GCF_000334155.1_L1/GCF_000334155.1_L1_protein.faa.gz

ftp://ftp.ncbi.nlm.nih.gov/genomes/all/GCF_000468755.1_OprofF0195v1.0/GCF_000468755.1_OprofF0195v1.0_protein.faa.gz

ftp://ftp.ncbi.nlm.nih.gov/genomes/all/GCF_000685235.1_SOAPdenovo_v1.05/GCF_000685235.1_SOAPdenovo_v1.05_protein.faa.gz

ftp://ftp.ncbi.nlm.nih.gov/genomes/all/GCF_001263175.1_ASM126317v1/GCF_001263175.1_ASM126317v1_protein.faa.gz

ftp://ftp.ncbi.nlm.nih.gov/genomes/all/GCF_001428765.1_Root79/GCF_001428765.1_Root79_protein.faa.gz

ftp://ftp.ncbi.nlm.nih.gov/genomes/all/GCF_000763315.1_ASM76331v1/GCF_000763315.1_ASM76331v1_protein.faa.gz

ftp://ftp.ncbi.nlm.nih.gov/genomes/all/GCF_000741325.1_Bifpse_sub.pse/GCF_000741325.1_Bifpse_sub.pse_protein.faa.gz

ftp://ftp.ncbi.nlm.nih.gov/genomes/all/GCF_000598065.1_Brdraft1/GCF_000598065.1_Brdraft1_protein.faa.gz

ftp://ftp.ncbi.nlm.nih.gov/genomes/all/GCF_000015585.1_ASM1558v1/GCF_000015585.1_ASM1558v1_protein.faa.gz

ftp://ftp.ncbi.nlm.nih.gov/genomes/all/GCF_000374525.1_ASM37452v1/GCF_000374525.1_ASM37452v1_protein.faa.gz

ftp://ftp.ncbi.nlm.nih.gov/genomes/all/GCF_001293525.1_ASM129352v1/GCF_001293525.1_ASM129352v1_protein.faa.gz

ftp://ftp.ncbi.nlm.nih.gov/genomes/all/GCF_000026045.1_ASM2604v1/GCF_000026045.1_ASM2604v1_protein.faa.gz

ftp://ftp.ncbi.nlm.nih.gov/genomes/all/GCF_000425585.1_ASM42558v1/GCF_000425585.1_ASM42558v1_protein.faa.gz

ftp://ftp.ncbi.nlm.nih.gov/genomes/all/GCF_000196355.1_ASM19635v1/GCF_000196355.1_ASM19635v1_protein.faa.gz

ftp://ftp.ncbi.nlm.nih.gov/genomes/all/GCF_000012685.1_ASM1268v1/GCF_000012685.1_ASM1268v1_protein.faa.gz

ftp://ftp.ncbi.nlm.nih.gov/genomes/all/GCF_000421045.1_ASM42104v1/GCF_000421045.1_ASM42104v1_protein.faa.gz

ftp://ftp.ncbi.nlm.nih.gov/genomes/all/GCF_000014445.1_ASM1444v1/GCF_000014445.1_ASM1444v1_protein.faa.gz

ftp://ftp.ncbi.nlm.nih.gov/genomes/all/GCF_000972865.1_ASM97286v1/GCF_000972865.1_ASM97286v1_protein.faa.gz

ftp://ftp.ncbi.nlm.nih.gov/genomes/all/GCF_000332215.1_ASM33221v1/GCF_000332215.1_ASM33221v1_protein.faa.gz

ftp://ftp.ncbi.nlm.nih.gov/genomes/all/GCF_000299895.1_ASM29989v1/GCF_000299895.1_ASM29989v1_protein.faa.gz

ftp://ftp.ncbi.nlm.nih.gov/genomes/all/GCF_000718095.1_ASM71809v1/GCF_000718095.1_ASM71809v1_protein.faa.gz

ftp://ftp.ncbi.nlm.nih.gov/genomes/all/GCF_000466985.1_ASM46698v1/GCF_000466985.1_ASM46698v1_protein.faa.gz

ftp://ftp.ncbi.nlm.nih.gov/genomes/all/GCF_000455265.1_Pa_GD11/GCF_000455265.1_Pa_GD11_protein.faa.gz

ftp://ftp.ncbi.nlm.nih.gov/genomes/all/GCF_000512895.1_ASM51289v1/GCF_000512895.1_ASM51289v1_protein.faa.gz

ftp://ftp.ncbi.nlm.nih.gov/genomes/all/GCF_000468475.2_Amad2/GCF_000468475.2_Amad2_protein.faa.gz

ftp://ftp.ncbi.nlm.nih.gov/genomes/all/GCF_000970285.1_ASM97028v1/GCF_000970285.1_ASM97028v1_protein.faa.gz

ftp://ftp.ncbi.nlm.nih.gov/genomes/all/GCF_000264495.1_ASM26449v1/GCF_000264495.1_ASM26449v1_protein.faa.gz

ftp://ftp.ncbi.nlm.nih.gov/genomes/all/GCF_000466565.1_ASM46656v1/GCF_000466565.1_ASM46656v1_protein.faa.gz

ftp://ftp.ncbi.nlm.nih.gov/genomes/all/GCF_000314575.1_ASM31457v1/GCF_000314575.1_ASM31457v1_protein.faa.gz

ftp://ftp.ncbi.nlm.nih.gov/genomes/all/GCF_001424905.1_Root112D2/GCF_001424905.1_Root112D2_protein.faa.gz

ftp://ftp.ncbi.nlm.nih.gov/genomes/all/GCF_000803065.1_ASM80306v1/GCF_000803065.1_ASM80306v1_protein.faa.gz

ftp://ftp.ncbi.nlm.nih.gov/genomes/all/GCF_000427785.1_ASM42778v1/GCF_000427785.1_ASM42778v1_protein.faa.gz
